# Supplementary material for: PDIM drives the transition from repairable to catastrophic EsxA-mediated vacuole membrane damage during Mycobacterium marinum infection
Source: PLoS Pathog. 2026 Oct 21;22(10):e1014721. doi: 10.1371/journal.ppat.1014721 (PMC13712727; doi:10.1371/journal.ppat.1014721)

raw image for Figure S1a: western blot anti-Mpt32 on bacterial lysates

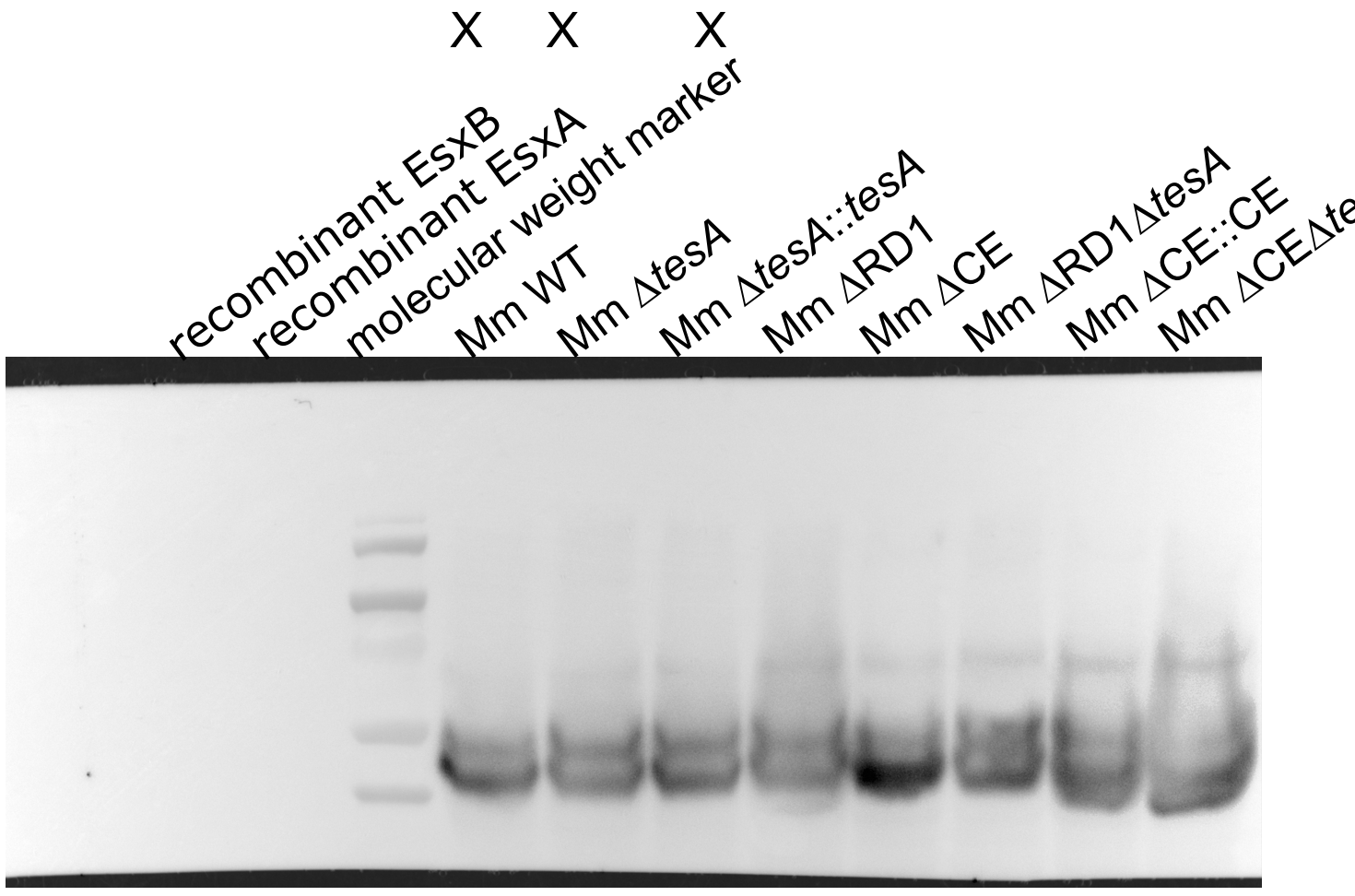

raw image for Figure S1a: western blot anti-GroEL on bacterial lysates

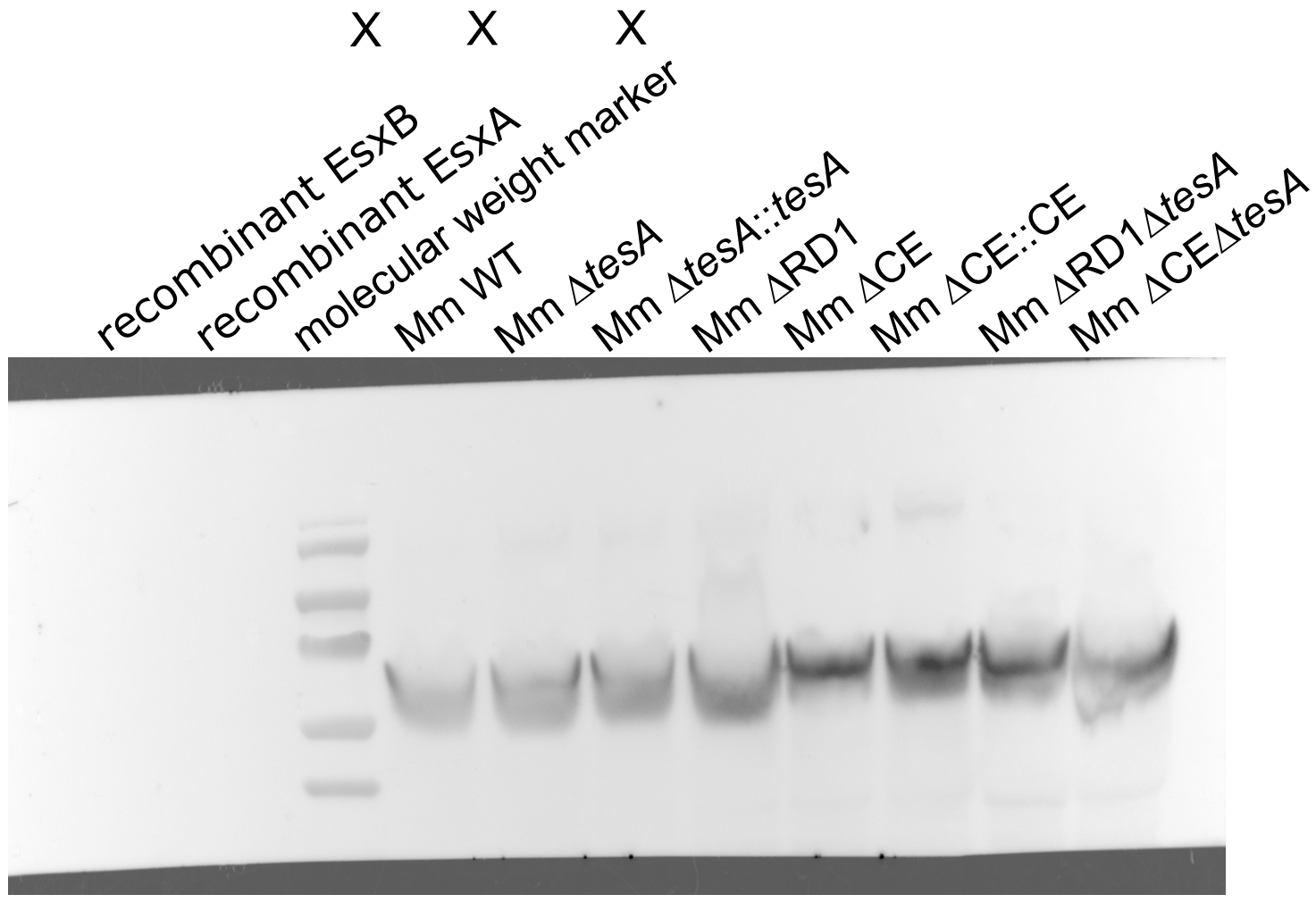

raw image for Figure S1a: western blot anti-EsxA on bacterial lysates

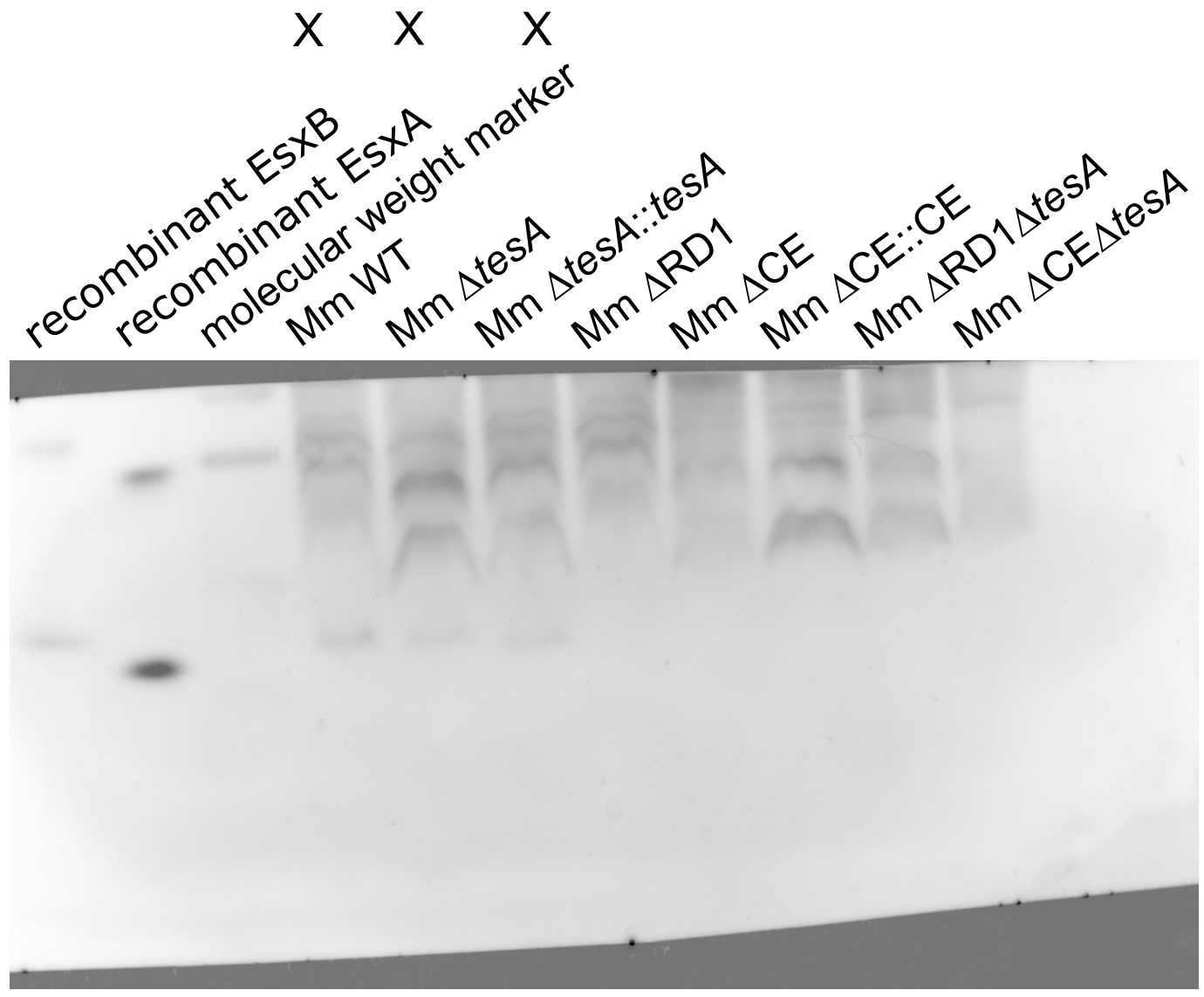

raw image for Figure S1a: western blot anti-EsxB on bacterial lysates

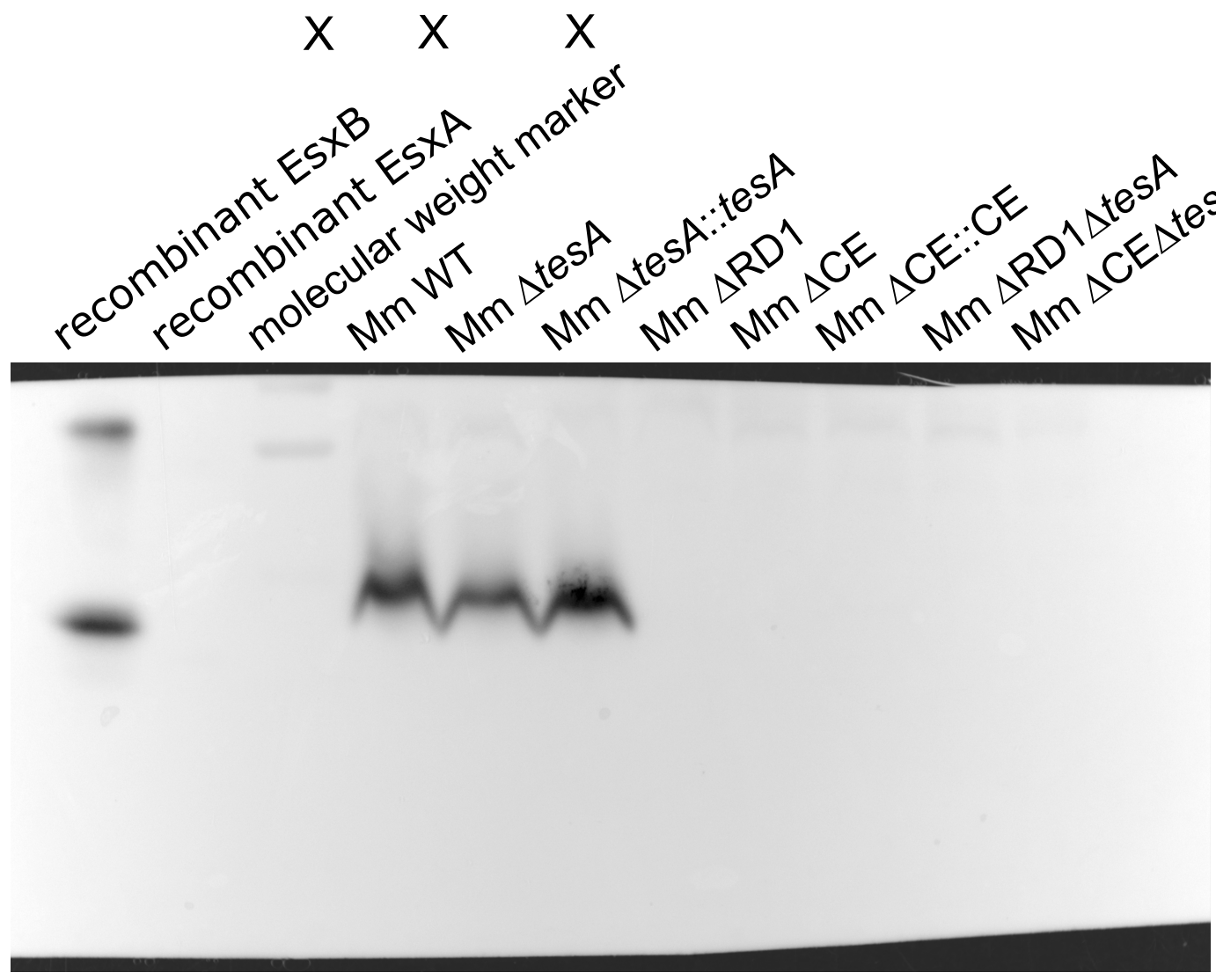

raw image for Figure S1a: western blot anti-EsxA (prolonged exposition) on bacterial lysates

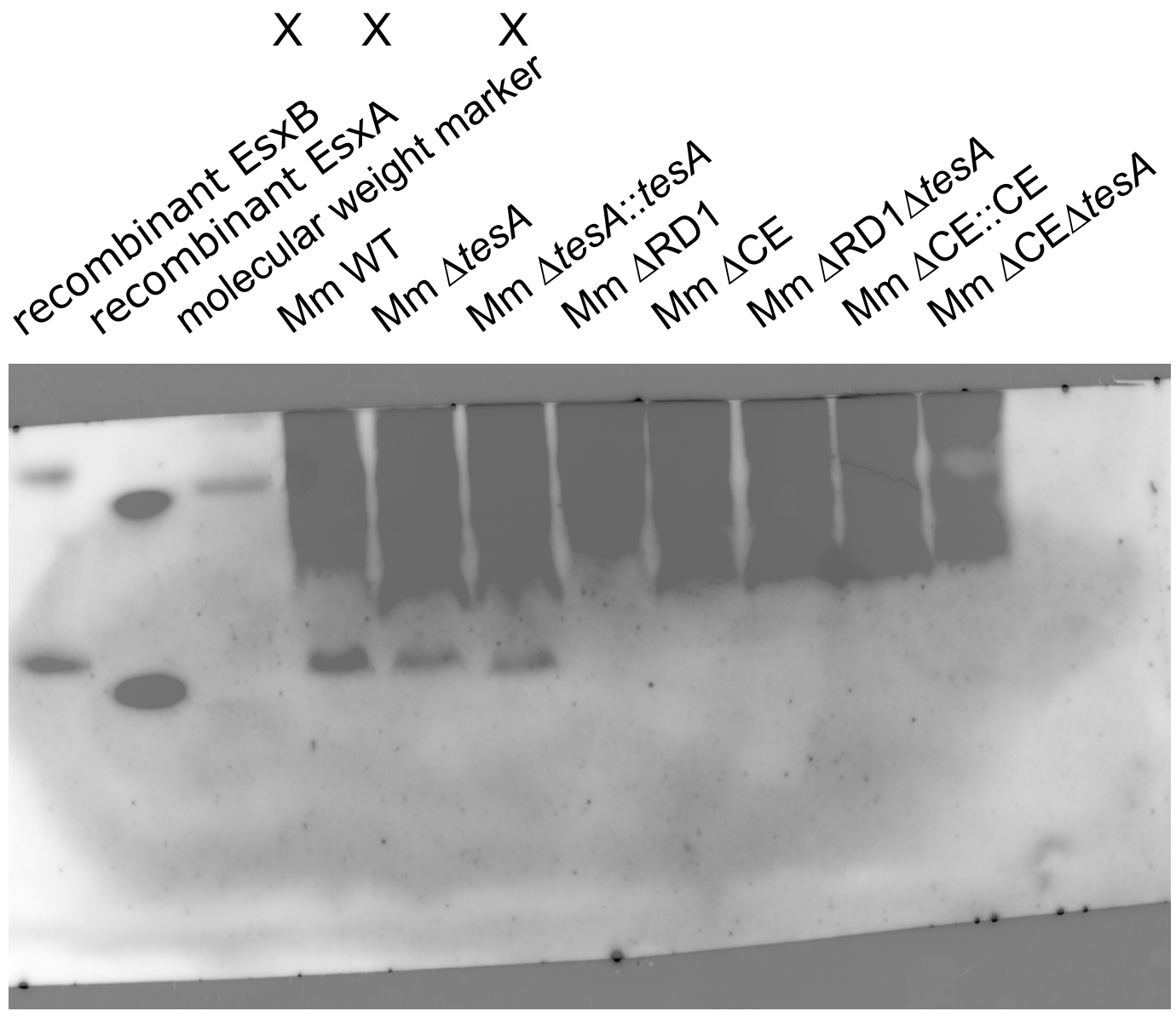

raw image for Figure S1a: western blot anti-EsxB (prolonged exposition) on bacterial lysates

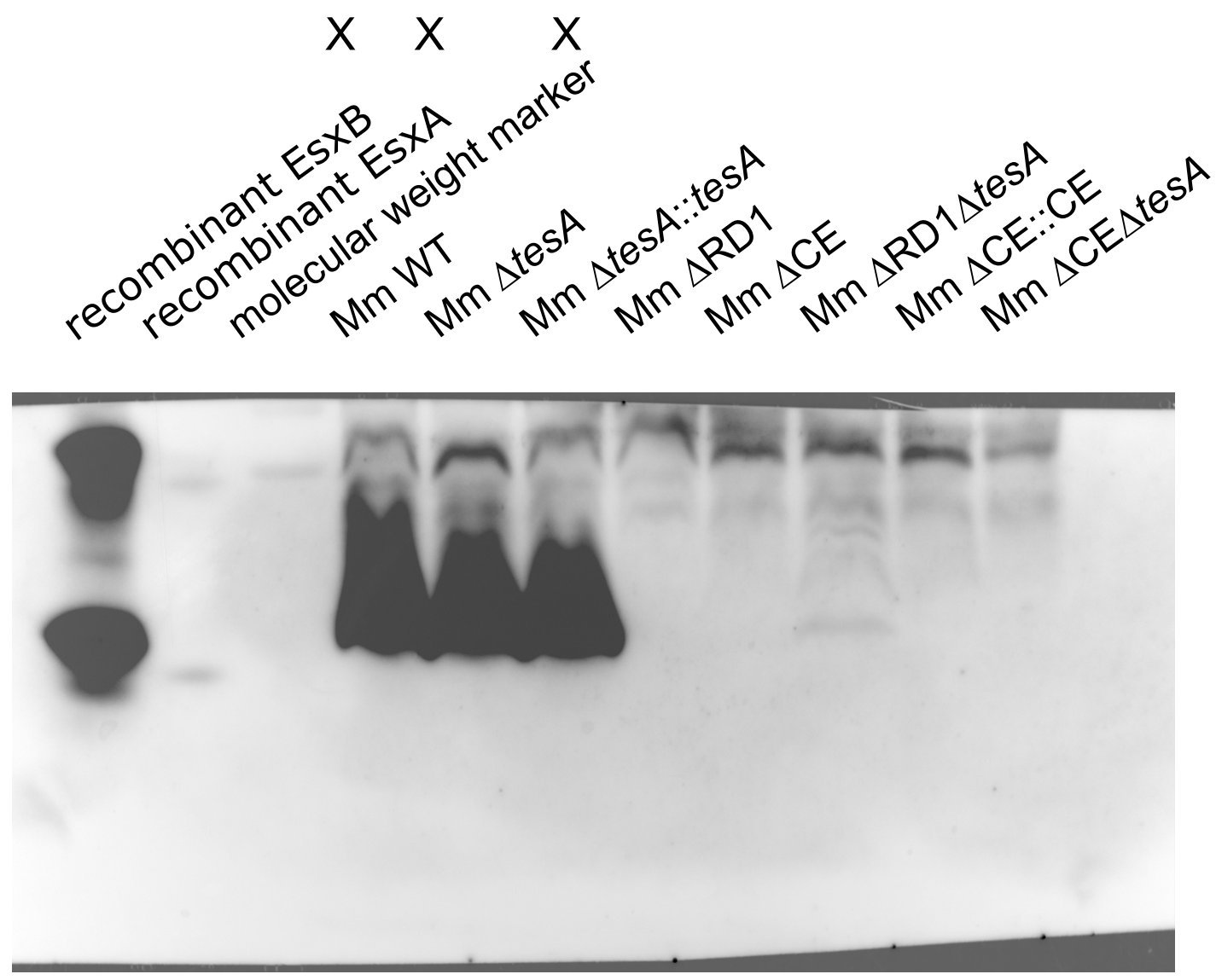

raw image for Figure S1a: western blot anti-Mpt32 on culture filtrates

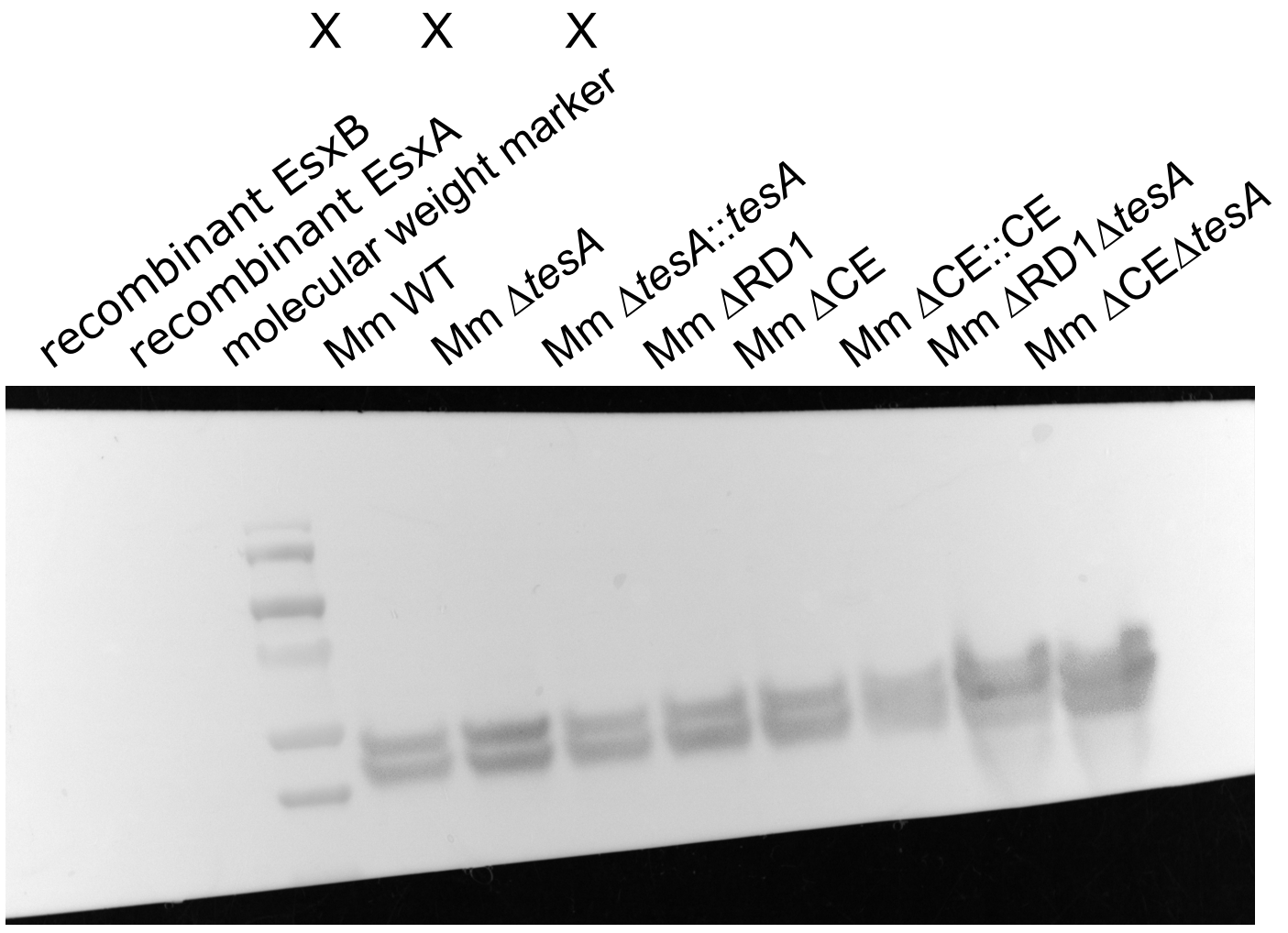

raw image for Figure S1a: western blot anti-GroEL on culture filtrates

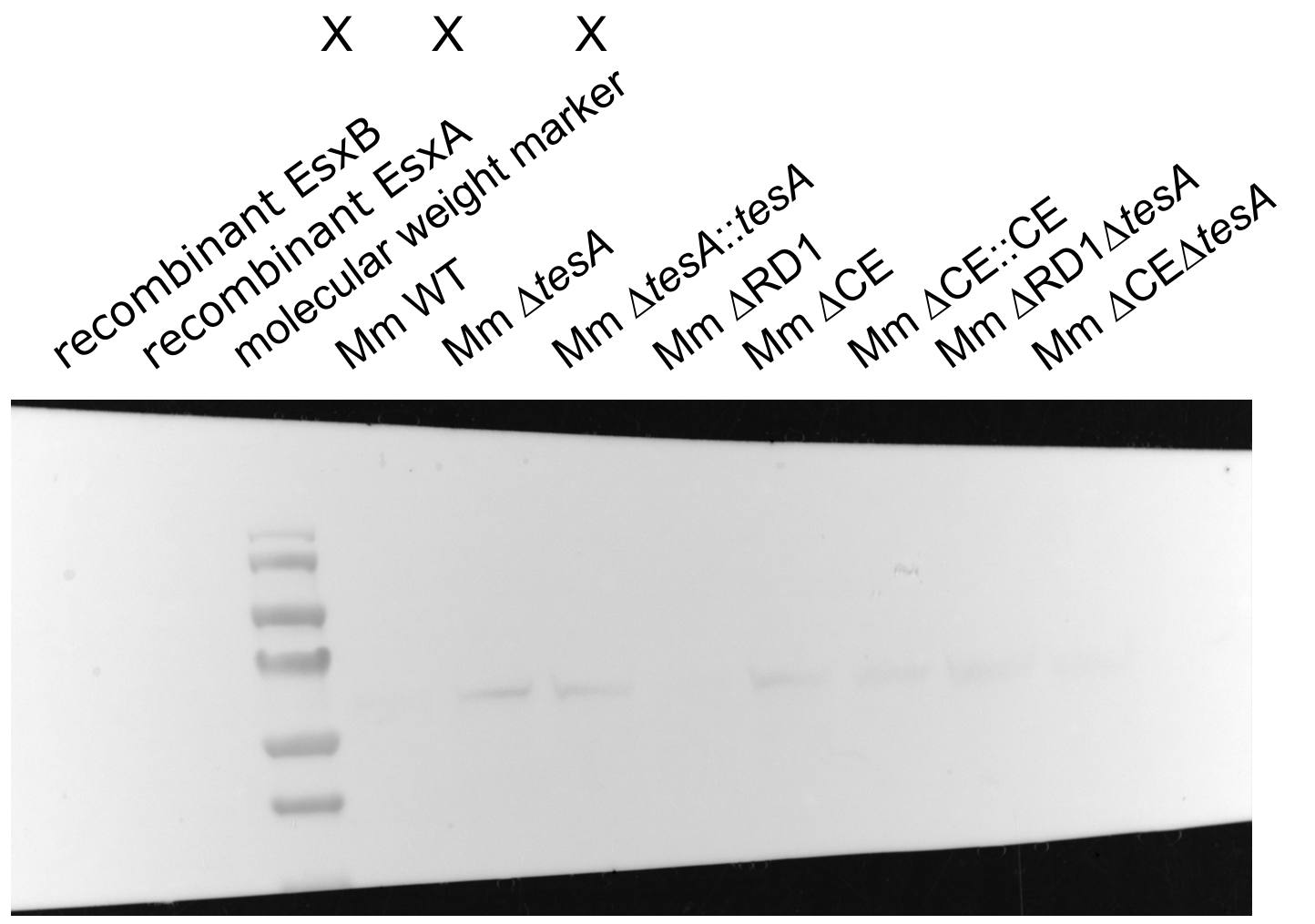

raw image for Figure S1a: western blot anti-EsxA on culture filtrates

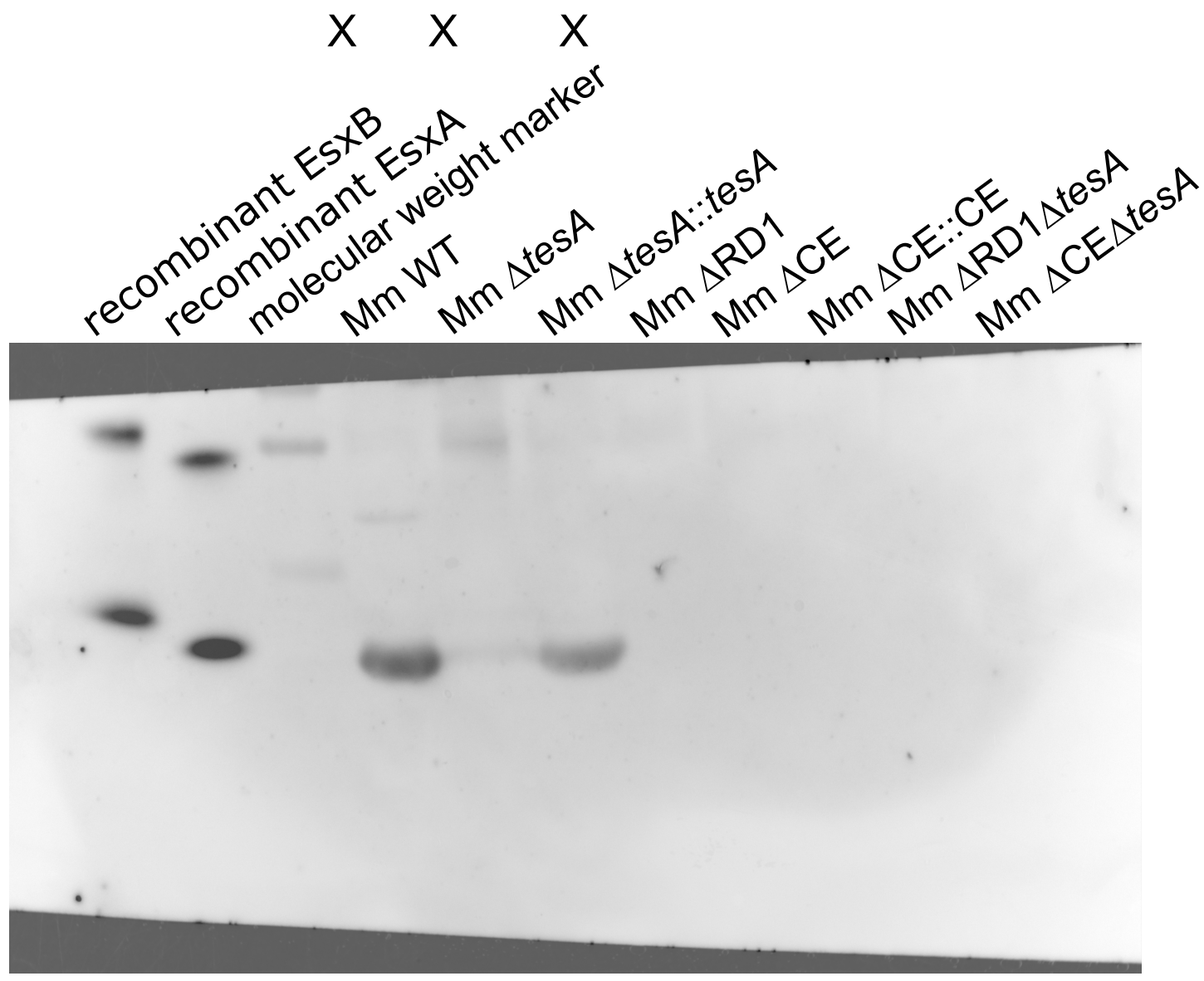

raw image for Figure S1a: western blot anti-EsxB on culture filtrates

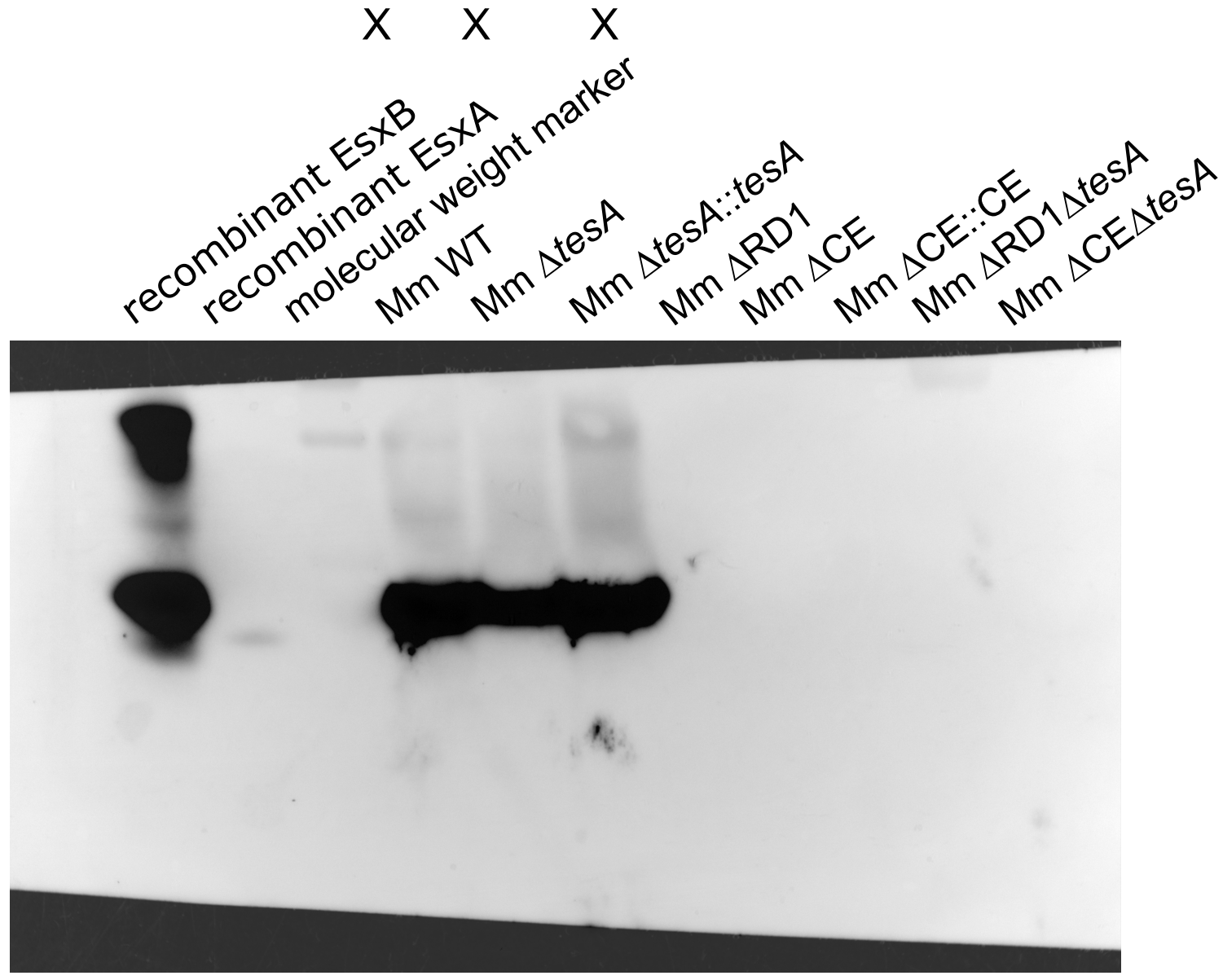

raw image for Figure S1a: western blot anti-EsxA (prolonged exposition) on culture filtrates

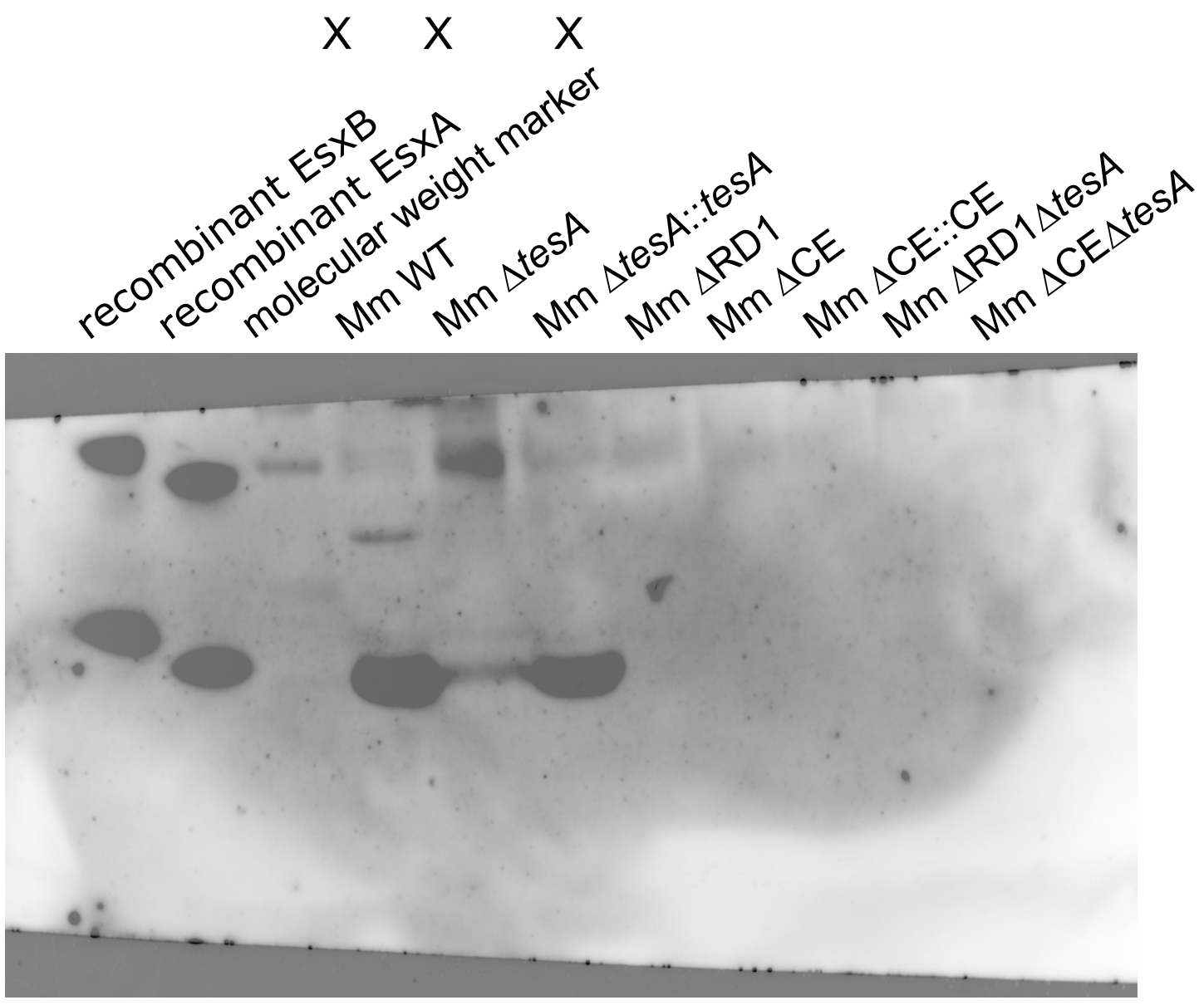

raw image for Figure S1a: western blot anti-EsxB (prolonged exposition) on culture filtrates

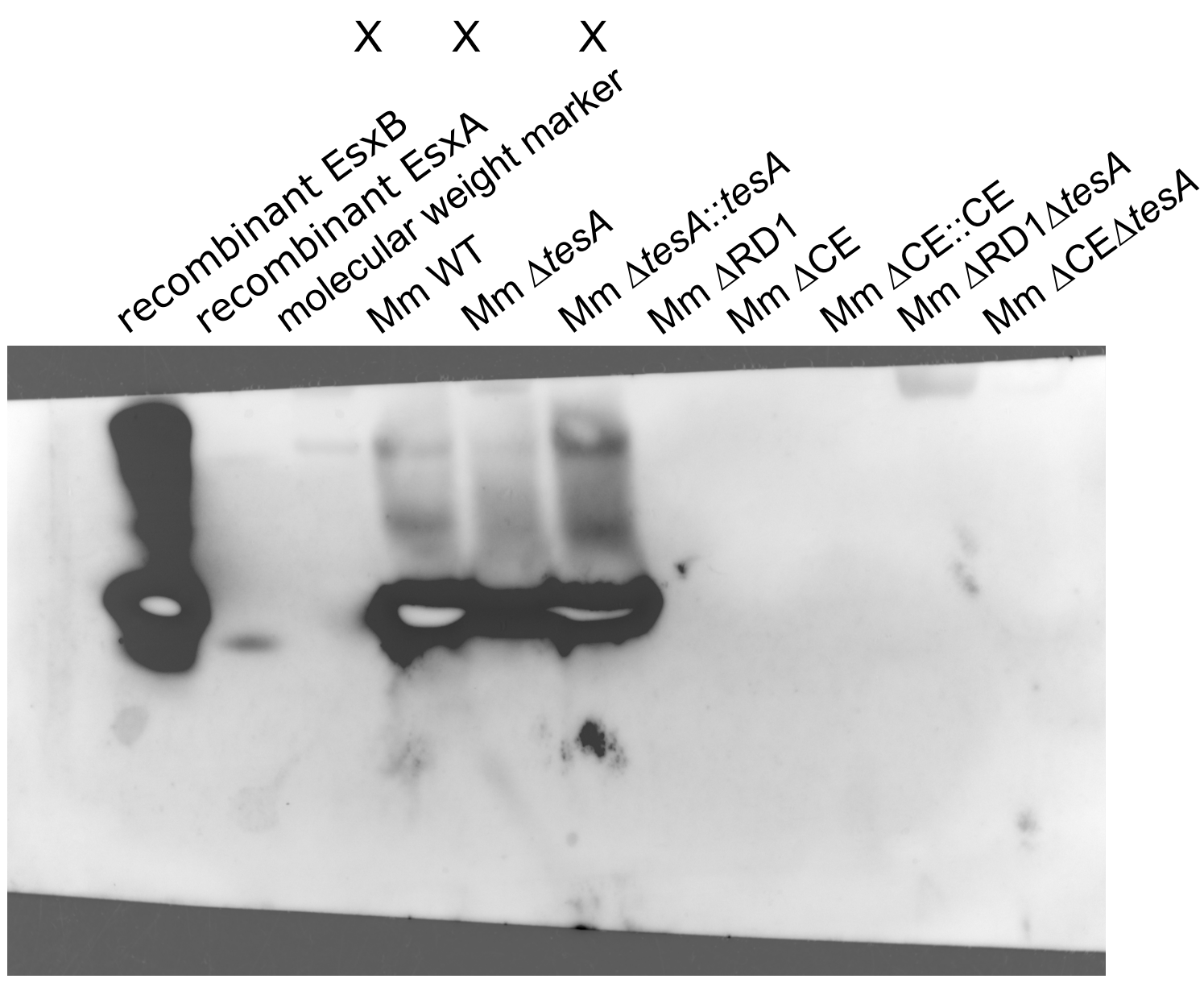

Supplement: Supplementary file 1 [file ppat.1014721.s009.pdf]
