# Supplementary material for: PDIM drives the transition from repairable to catastrophic EsxA-mediated vacuole membrane damage during Mycobacterium marinum infection
Source: PLoS Pathog. 2026 Oct 21;22(10):e1014721. doi: 10.1371/journal.ppat.1014721 (PMC13712727; doi:10.1371/journal.ppat.1014721)
Supplement: Supplementary file 2 [file ppat.1014721.s011.pdf]

Reviewers' Comments:

Reviewer's Responses to Questions

Part I - Summary

Please use this section to discuss strengths/weaknesses of study, novelty/significance, general execution and scholarship.

Reviewer #1: The manuscript by Michard et al is a great piece of work that attempts to decipher the respective contributions of ESX-1 effectors and the PDIM lipids during infection by Mycobacterium marinum. The authors succesfully use host reporters in two infection models to show how these factors act at different stages of the dynamic process of damage and rupture of the MCV. Overall the work is solid, although I have a few major points that could be improved to ensure the strength of the work. Importantly, from a technical point of view, given that they are trying to show co-localization with different cell markers, it becomes indispensable that the authors change their images to colour-blind safe combinations, I have also stated this under Minor issues.

I think for the field, this manuscript clearly shows how there is a difference between EsxA-induced damage that is amplified by PDIM, which is a key knowledge gap. I think further dissection of how it works mechanistically is something that can be tackled in the future.

Reviewer #2: This manuscript thoroughly addresses an important open question regarding the contribution of PDIM to membrane damage during Mm infection. The authors use genetic perturbations in both the bacteria and the host to investigate the sequential contributions of EsxA and PDIM to cytosolic access by Mm. The manuscript is clearly written and appropriately references recent literature, providing a detailed overview of the current understanding in the field and clearly explaining how this study contributes to resolving several outstanding questions. A few minor improvements could further enhance clarity for readers in certain sections, as outlined in the minor comments below.

Reviewer #3: The study by Michard et al demonstrates the synergy between EsxA/B and PDIM-driven membrane damage. The authors use state-of-the-art live cell imaging approaches in combination with an extensive series of Mm deletion mutants and/or host cell mutants to show the differential impact of the lack of EsxA/B, PDIM/PGL or both on host cell ESCRT and autophagy responses. Overall the work significantly advances the knowledge in the field generated by the Soldati lab in the Dictyo/Mm model and other labs in the Mtb/macrophage model.

We thank each reviewer for their positive assessment of our work and for recognizing the significance of this study in deciphering the mechanism of MCV membrane damage and for its general relevance to the host-pathogen interaction field. We appreciate the constructive suggestions to improve the manuscript and figures. We apologise for not having used colour-blind safe combinations for our representative images, as mentioned by reviewers #1 and #2. We have now corrected this issue. We have addressed each of the reviewers' comments below and have revised the manuscript accordingly.

Part II – Major Issues: Key Experiments Required for Acceptance

Please use this section to detail the key new experiments or modifications of existing experiments that should be absolutely required to validate study conclusions.

Generally, there should be no more than 3 such required experiments or major modifications for a "Major Revision" recommendation. If more than 3 experiments are necessary to validate the study conclusions, then you are encouraged to recommend "Reject".

Reviewer #1: The first main issue I see with the manuscript in its current version is the use of diverse host markers, especially for damage and rupture and later on, autophagy, but they never show co-localization with a MCV marker, making me question how can the authors truly differentiate between cytosolic and vacuolar Mm.

We thank the reviewer for raising this point. We agree that it is important to clearly differentiate between cytosolic and vacuolar bacteria. In our study, we only used well-established reporters for which solid and reproducible evidence of their association with cytosolic or vacuolar Mm is available [1-8]. The visualization of the ESCRT reporter at the bacteria necessarily involves the presence of a membrane, as ESCRT by nature can only interact with a biological membrane [9]. The machinery for autophagy-mediated repair is recruited to damaged MCVs and prevents escape [1-6]. Xenophagy captures and eliminates only cytosolic bacteria that are accessible to Plin coating [5-8]. These reporters are also extensively used in mammalian systems by other groups that clearly demonstrated their association with cytosolic or vacuolar Mtb [10-13]. In our study, we can therefore conclude that Mm associated with Vps32, Atg8A, lysenin, NDP52, LC3b and ubiquitin are bacteria contained in an MCV at different stages of the membrane damage-repair cycle, while Plin-positive bacteria are majorly exposed to the cytosol.

Second, it is well established in the field of membrane damage and rupture during bacterial pathogenesis, that a good marker for rupture is lysenin, which the authors use; but for rupture they did not use any of the established rupture markers, such as galectin (3 or 8) which are crucial to differentiate damaged vacuoles (lysenin positive, galectin negative) from ruptured vacuoles (galectin positive). Such experiments I would consider crucial to differentiate between the two stages and would validate their Plin experiments, as this only binds the myco cell wall but does not report for the MCV membrane itself.

We thank the reviewer for this alternative idea to differentiate damaged vacuoles from fully ruptured vacuoles. First, we have to admit that our main host model, Dd, has some limitations. Dd synthesises inositol-phosphorylceramides and not sphingomyelin and therefore we cannot use lysenin as a specific reporter for damaged vacuoles. Dd has functional homologs of cytosolic galectins but, unfortunately, they do not recognize host glycans like mammalian galectins [14]. We are therefore limited in this system, and this is why Plin was used as a marker of ruptured vacuoles. We agree that Plin binds directly to the Mm cell wall, but it has been well characterized that Plin reaches the bacteria only once the vacuole is severely ruptured [7]. The combination of our Plin recruitment results during infection and the *in vitro* Plin-binding assay clearly demonstrate that Mm Δ*tesA* does not rupture its vacuole, as it does not associate with Plin during infection.

Concerning our mammalian phagocyte system, the murine microglial BV-2 cells, we tried, as suggested by the reviewer, the triple colocalization of Mm, GFP-lysenin and an anti-Gal3. Although we used the anti-Gal3 antibody used in multiple studies of *Mycobacterium tuberculosis* infection in human cells [13, 15], the quality of our Gal-3 staining was not good enough to allow a hight-content hight-throughput computer-assisted analysis. We guess that the insufficient quality of the immunostaining comes from specific murine BV-2 associated limitations. We are nevertheless confident that the combined analyses of lysenin, NDP52, LC3b

recruitment and ubiquitination presented in the manuscript are sufficient to demonstrate that Mm Δ*tesA* produces intermediate levels of damage to its MCV in BV-2 microglial cells.

In addition, the authors could complement their plate reader experiments with CFU counting, especially considering that they are not tracking individual bacteria overtime by microscopy.

We thank the reviewer for this suggestion. We agree that plate reader measurements could be complemented by another approach, such as CFU, to determine bacteria burden. However, Mycobacteria are famous for their excessive clumpiness, which makes them difficult to cultivate and makes CFU measurements unreliable. We took advantage of measuring bacteria burden by following the bacteria fluorescence with a plate reader, as it has been clearly demonstrated that there is a linear correlation between CFU and fluorescence or luminescence (see below the Figure S4 from Trofimov et al. [16]).

We have now added a sentence about the linear correlation in the Materials & Methods section, infection part (lines 805-806): "The measurement of bacteria green fluorescence has been shown to correlate linearly with Colony-Forming Units [66], making it a reliable indicator of bacteria burden."

Moreover, to confirm the initial intracellular growth defect of the Mm mutants and validate the robustness of the plate reader measurements, we also performed high-content microscopy (Fig 1c,d). After segmentation, the bacteria area was tracked overtime by microscopy and confirmed the plate reader results. We thus used primarily plate reader measurements in the following experiments of the manuscript for reasons of material availability and cost.

Supplementary figure 4
Trofimov et al.

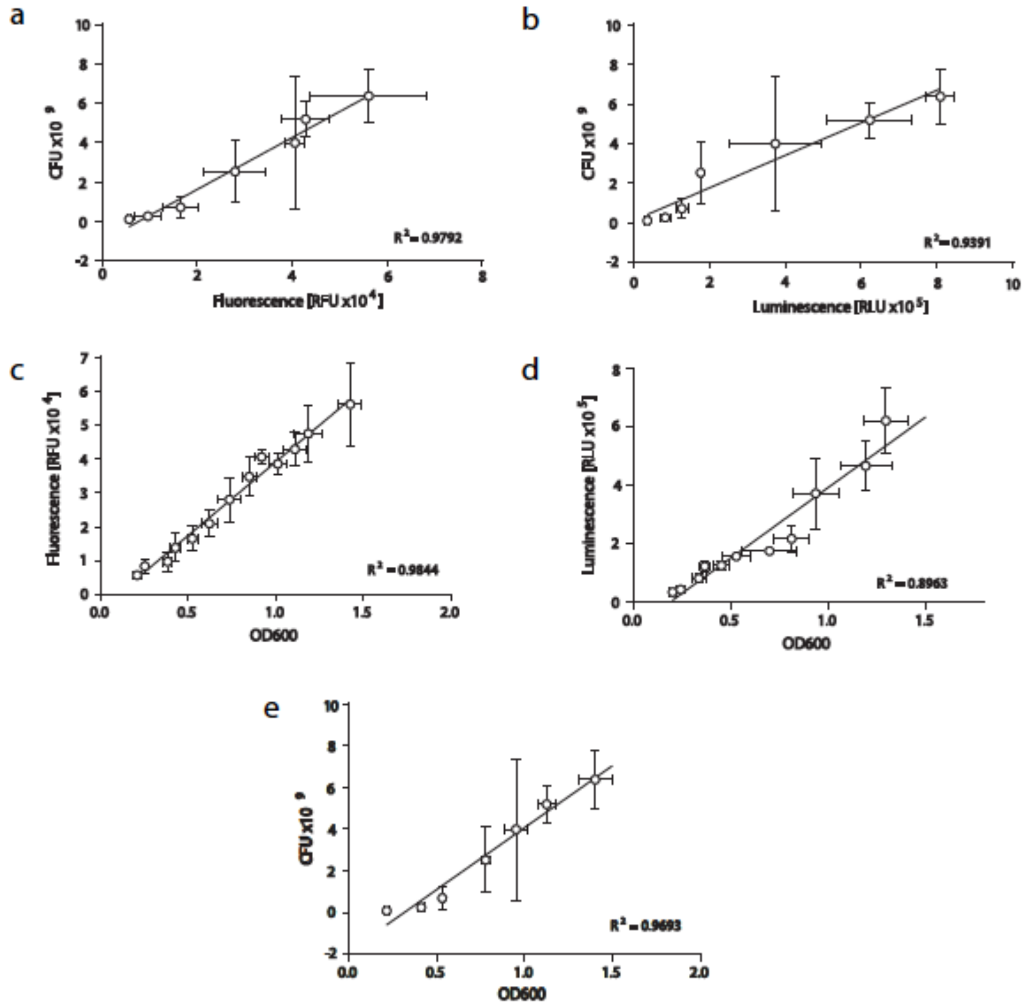

Trofimov et al, 2018, Figure S4. Determination of the correlation between CFU counting, OD, bioluminescence and fluorescence measurements in *M. marinum* cultures. The relationships between CFU/RFU (A), CFU/RLU (B), RFU/OD (C), RLU/OD (D), and CFU/OD (E) were characterized and coefficients of linear correlation were calculated. *M. marinum* strains either without reporter or expressing Lux or GPF were cultured as described before. The overnight cultures were diluted 10-fold into the same, fresh medium in order to obtain an OD600 of 0.2. Samples were taken at regular intervals during growth as indicated in the graphs. After incubation at 32°C, colonies on the plates were counted to determine the number of CFU.

Reviewer #2: (No Response)

Reviewer #3: 1) There seems to be a discrepancy between the growth of the Mm ΔtesA mutant in Dd (Fig. 1d) and BV2 cells (Fig. 5b) which needs to be addressed

We thank the reviewer for pointing out this difference. We agree that the intracellular growth phenotype of Mm Δ*tesA* is not identical in the two host models. However, the hierarchy of the Mm mutants' growth attenuation is conserved between the two hosts. Importantly, Mm Δ*tesA* showed an intermediate phenotype in both systems, being not as strongly attenuated as the other Mm mutants compared with Mm WT. In BV-2 cells, even if the difference between the growth of Mm WT and Mm Δ*tesA* was not statistically significant, Mm Δ*tesA* consistently ranked below Mm WT in each biologically replicated experiment. Moreover, the additive attenuation observed for the double mutants Mm ΔRD1Δ*tesA* and ΔCEΔ*tesA* confirmed that the loss of PDIM contributed to growth attenuation in BV-2 cells.

Please, also note that several experimental and biological differences between the two infection systems likely contribute to this difference. The experiments were performed at different temperatures, with BV-2 infections incubated at a more optimal temperature for Mm (32°C) compared to Dd infections (25°C). We can also not exclude that the small differences in host-cell membrane composition between Dd and BV-2 cells influenced the MCV properties and nutrient availability for Mm Δ*tesA*. We therefore do not interpret the difference between the two models as evidence for a different role of PDIM. We consider that the Mm Δ*tesA* growth amplitude is rather host- and experimental-context dependent.

We have now changed the Results section of the manuscript to more explicitly explain this difference (lines 411-416): "The difference in growth attenuation of Mm Δ*tesA* between Dd and BV-2 may result from differences in temperature, as BV-2 infections were performed at 32 °C, the optimal growth temperature for Mm, contrary to Dd experiments performed at 25°C, the maximal temperature for Dd. The additive attenuation observed for Mm ΔRD1Δ*tesA* and Mm ΔCEΔ*tesA* nevertheless suggests that the *tesA* deletion alone slightly attenuates the growth of Mm in microglial cells."

2)There is strong evidence that ER-mediated membrane repair is of particular importance for membrane repair after infection with either Mtb or Mm. How the mutants affect recruitment of ER-membrane repair proteins to the MCV should be included to complete the picture

We thank the reviewer for this interesting idea. We agree that ER-associated membrane repair is now established as an important pathway involved during membrane repair of Mtb or Mm vacuoles, in macrophages and Dd. However, we consider that analysis of ER-associated repair would provide an extension of our study rather than information about the mechanism described in the present study. Our data show that EsxA-dependent damage is associated with recruitment of both ESCRT and autophagy machineries whereas PDIM is required for progression towards extensive damage and cytosolic access. Our main message is the sequential contribution of EsxA and PDIM to MCV damage, and an investigation of ER-associated repair is not required to establish this conclusion.

We nevertheless included new sentences in the Discussion section to empathize this interesting suggestion (lines 519-523): "Moreover, ER-mediated membrane repair through OSBP proteins and membrane wound "plugging" by stress granules have been recently identified as important pathways involved during MCV membrane damage/repair [21, 28, 29]. Analysing the contribution of EsxA and PDIM sequential action on both pathways will help in characterising more precisely their respective engagement during Mtb/Mm infection."

Part III – Minor Issues: Editorial and Data Presentation Modifications

Please use this section for editorial suggestions as well as relatively minor modifications of existing data that would enhance clarity.

Reviewer #1: 1) I think it is fundamental for the authors to change their figures to a colour blind-safe combination, and avoid all green-red combinations to show co-localizations. This is especially critical for figures with low magnification like 1c, 2g, etc, but it should be implemented in all of them.

We thank the reviewer for mentioning this important point. We have now modified each representative microscopy image in all figures to avoid the green-red combination and to use a colour-blind-friendly combination (magenta-green).

2) It is interesting to this reviewer that in Figure 3c there seems to be a fixed percentage of bacteria accessing the cytosol up to 36 hpi. Can the authors check if there is replication in the cytosol? Is this the total subpopulation that escapes to the cytosol?

We thank the reviewer for this interesting observation. Yes, Mm is known to replicate in the host cytosol, as previously demonstrated in several studies [3, 5, 6, 17] as well as here, using Dd Δ*atg1*, which allows earlier and more efficient escape to the cytosol and subsequent unrestricted growth in the absence of xenophagy. Unfortunately, in the experiment corresponding to Fig 3d (previously Fig 3c), we cannot specifically distinguish the population able to replicate in the host cytosol. The plateau observed for the proportion of Plin-positive bacteria should not be interpreted as evidence that the cytosolic population is not replicating, but rather as a result of the heterogeneity of events happening at the same time. Indeed, even if we spinoculate the infections to synchronize them, the exact timing at which each MCV is engaged in various steps of the membrane damage/repair cycle is heterogeneous, and the bacteria are progressively released into the host cytosol. However, in parallel, xenophagy will recapture some of those released earlier. Cell-to-cell spreading by ejection and upon host cell death are also events that influence the percentage of Plin-positive Mm, as these bacteria will become extracellular or be recaptured by naïve bystander cells, triggering a new infection cycle. The overall percentage of bacteria in the cytosol results from the global dynamics of the infection.

3) Regarding data presentation, it would be ideal to include the time post infection in all figure panels in which representative microscopy images are presented. I detected that in some figure legends this is not stated, such as 2a-b-c-d-e-f, 3a-b-c, 4g, 5c-d-e-f (here the authors state that cells were fixed at 5 and 24 hpi, but it is not stated which time point is shown in the selected images).

We thank the reviewer for pointing out this missing information. We have revised the manuscript and the figures. We included the time post-infection for each representative microscopy image directly on the figure panels or in the corresponding figure legend.

4) In line 432, the authors state “Ubiquitin accumulation is an early sign of membrane damage and persist until the compartment is repaired or cleared”, please provide the corresponding citation.

We thank the reviewer for highlighting this statement without a reference. After careful literature review, we found that our statement was not appropriate. Ubiquitin has been identified as an early indicator of MCV membrane damage in several studies, preceding LC3b recruitment [5, 6, 11, 18]. Different works also demonstrated that persistent or unresolved membrane damage produces sustained or even enhanced ubiquitination at the MCV in Mtb/macrophage model as well as in Dd/Mm system [5, 6, 19, 20]. However, there is no clear evidence that ubiquitin remains continuously on an individual MCV until that precise MCV is repaired or cleared. We have revised the manuscript accordingly to the literature and replaced the previous sentence in the Results section by (lines 444-447): “Ubiquitination of bacterial and host proteins is an early MCV damage-associated signal [11, 13, 26, 68, 69]. When membrane damage is not efficiently resolved, ubiquitination persists or accumulates at the MCV, ultimately promoting xenophagic clearance [11, 13, 70, 71].”

5) I found Figure S3 quite confusing, although is supposed to show individual tracks of MCVs overtime, some of them are merged. It would make much more sense to keep them individual. Moreover, for MCVs tracked that are appearing and disappearing from the field of view (from what I can interpret), how did the authors ensured that they are from the same cells?

We thank the reviewer for this constructive suggestion. We agree that Fig S3 could be clearer. We have modified the figure as suggested to display separate individual tracks for the 140 independent MCVs.

The MCVs represented in Fig S3 were manually tracked through live imaging based on their spatial position and fluorescence signal, allowing us to follow the same MCV over consecutive time points. The light grey representation corresponds effectively to MCVs disappearing and reappearing in the field of view. We considered that it was the same MCV that reappeared following two scenarios: First, in some movies, we had a focus issue at one time point. This issue allowed us to track the individual cells but not the corresponding MCV. The second scenario is that in some specific cases, the cell was positioned at the border of the field. We could follow that it was the same cell, but as the MCV was moving inside the cell, the MCV could move outside the field of view. In both cases, we represented the specific time points in light grey on the track representation and then continued MCV tracking with the reporters. In case of doubt about the identification of an MCV, a new track was represented to ensure the robustness of the tracking. We have now clarified this point in the figure legend (lines 1368-1370): “Light grey corresponds to transiently invisible MCVs, either because the specific MCV moved outside the field of view at that time point or because of a focus issue.”

Reviewer #2: For the experiments shown in Figures 1d and 5b, why did the measurements end at 48h rather than 72h? Was this due to a technical limitation of the imaging approach, or was the 48h endpoint selected intentionally? If so, the rationale for changing the endpoint should be clarified. Although extending the experiment may not alter the overall conclusion, it could further emphasize the observed differences and allow a more direct comparison with Figure 1b.

We thank the reviewer for noticing this difference. We agree that extending the experiments in Fig 1d and 5b to 72 hpi would have emphasized the observed differences and allowed a more direct comparison with Fig 1b. Unfortunately, due to technical limitations, these two experiments were monitored until 48 hpi. Fig1d represents the monitoring of Mm intracellular growth in Dd by high-content high-throughput microscopy. To analyse this experiment, we segmented the cells and the bacteria. Unfortunately, if this experiment is conducted until 72 hpi, Dd cells are too confluent, and we no longer succeeded in properly segmenting the cells and identifying the bacteria. We do not have these limitations with the plate reader experiments, as we measured the global green fluorescence from the entire well. In Fig 5b, we measured Mm intracellular growth in BV-2 cells by high-content high-throughput microscopy. 48 hpi is the last time point as after this time, host cell death

and Mm release becomes a dominant feature, precluding meaningful tracking. Sentences have been added to the Materials & Methods section to emphasize these technical limitations:

For the experiment corresponding to Fig 1d (lines 810-8011): “To monitor intracellular growth, the plates were imaged every 4 h until 48 hpi to avoid over-confluency of Dd and allow proper segmentation prior to analysis.”

For the experiment corresponding to Fig 5b (lines 818-820): “To monitor intracellular growth, the plates were imaged every 1.5 h until 48 hpi as after this time, host cell death and Mm release become a dominant feature, precluding meaningful tracking.”

In Figure 1c, the micrographs are difficult to interpret/see, particularly the smaller grayscale images. Including representative zoom ins would help readers to better understand the differences between conditions. More generally, for all colocalization images, it would be helpful to display the individual channels separately, as shown in Figures 2a–c, and to include zoom ins centered on the bacteria. Using a color-blind-friendly colors and avoiding gray where possible would further improve.

We thank the reviewer for this advice. We changed all the representative images to display colour-blind friendly pictures and replaced the single grey channel representation by colour images. All the images now display individual channels, as for Fig 2d, 2e and 2f, where we added a supplementary figure (Fig S4) to display them. We did not include more zoom-ins in the figures, as the pictures showing the precise reporter localization already had/were zoom-ins and the images without zoom-ins are intended to show the global bacteria load and not a specific detail.

In Figure 2, the authors show representative examples of the dynamic recruitment patterns of Vps32 and Atg8 to the WT and mutant Mm strains. It would be helpful to include a schematic summarizing these distinct recruitment events and grouping them according to their temporal patterns, either alongside the corresponding images or as an additional panel within the figure. The figure would also benefit from indicating the frequency of each event and clearly linking these categories to the quantification shown in Supplementary Figure 3. Although Supplementary Figure 3 contains multiple examples, it is currently difficult to relate them directly to the representative events shown in Figure 2. One possible solution would be to include the corresponding timeline bars from Supplementary Figure 3 beneath each representative example.

We thank the reviewer for these suggestions. Unfortunately, the results obtained from the double-expressing Dd cell lines are very heterogeneous, making the classification of events difficult. We created the Fig S3 to specifically represent the variety of events observed, as their heterogeneity made them impossible to classify when considering changes over time. We have now modified Fig S3b to display the percentage of the three categories of observed events at each time point (GFP-Vps32; mCherry-Atg8A; GFP-Vps32+mCherry-Atg8A), rather than only the global GFP-Vps32 or mCherry-Atg8A ratios. Therefore, Fig S3b now represents the frequency of each event over time. We have also added a note to Fig S3a to facilitate identification of the tracks from which the bacteria shown in the representative images from Fig 2a-f originate.

For Figures 3 and 4, again a graphical summary linking the different damage and repair markers to the authors' conclusions would help readers follow the proposed sequence of events. The authors could adapt the model presented in Figure 6 to illustrate Vps32, Atg8 and Plin recruitment as an indicator the damage stage. Including a schematic alongside these figures would make the interpretation of the different markers and their relationship to the reader more intuitive.

We thank the reviewer for this constructive suggestion. We have now added schematic representations in Fig 3 (Fig 3a) and in Fig 4 (Fig S6).

The authors state in line 348: “Importantly, the reduction in MCV damage resulted in confinement of Mm WT and thus phenocopied infection with the Mm ΔRD1 mutant.” It would be helpful to specify which data support this comparison and to include the corresponding figure reference at the end of the text.

We thank the reviewer for noticing the missing reference. We apologise as, in fact, it was misleading to combine the results included in this manuscript with the results from Perret et al. [2]. We have changed this paragraph to clarify the claims (lines 346-358):

“It was previously shown that the MCV membrane composition, and specifically the presence of sterol-rich microdomains, is critical for EsxA to induce membrane damage [20]. Perret et al demonstrated that deletion of the three Dd vacuolins, functional homologues of the mammalian microdomain organizer flotillins, or the addition to Dd WT cells of methyl-β-cyclodextrin, which depletes sterol from sterol-rich membranes, significantly decreased both MCV damage and the intracellular growth of Mm WT. Importantly, the reduction in MCV damage resulted in confinement of Mm WT and phenocopied infection with the Mm ΔRD1 mutant [20]. These conditions were used here to monitor the various Mm mutants. As expected, the growth of Mm WT was attenuated both in the Dd vacuolin mutant (Dd Δ*vacABC*) and in the presence of methyl-β-cyclodextrin (Dd + CD), compared to Mm WT growth in Dd (Fig 4a, 4b, 4c). Under conditions that disrupt sterol-rich microdomains, the growth of Mm ΔCE (Fig 4a), Mm Δ*tesA* (Fig 4b) and Mm ΔCEΔ*tesA* (Fig 4c) was not further attenuated. Because Mm Δ*tesA* induces EsxA-mediated damage, a stronger growth restriction would have been expected upon disruption of sterol-rich microdomains.”

One last concern is the level of EsxA secretion in the tesA mutant. Although the authors acknowledge this issue in the limitations section, it would be helpful to discuss it earlier. In particular, the authors could compare the tesA mutant with the complemented CE strain, which also appears to exhibit reduced EsxA secretion despite phenotypic complementation. Discussing the distinct damage/repair profiles observed between the wild-type, tesA and CE strain would strengthen the interpretation of the results.

We thank the reviewer for this suggestion. The level of secreted EsxA was variable between the different replicates, but we agree that in this example, Mm Δ*tesA* secreted less EsxA than Mm WT. Nevertheless, the quantity of EsxA secreted by Mm Δ*tesA* was higher than the one secreted by the complemented strain of Mm ΔCE. And importantly, the low level of EsxA secreted from complemented Mm ΔCE was sufficient to restore the virulence of the strain, meaning that the growth attenuation observed for Mm Δ*tesA* is plausibly not due to a low level of secreted EsxA. We agree that we could have commented the level of EsxA secretion between the Mm Δ*tesA* mutant and the Mm complemented ΔCE strain in the manuscript. We thus now added a sentence to the Results section to this purpose (lines 226-229): “Although EsxA secretion was reduced in the Mm Δ*tesA* mutant, it remained readily detectable, whereas phenotypic complementation of the Mm ΔCE mutant was achieved despite almost undetectable EsxA/EsxB levels. This argues that the reduced EsxA secretion in Mm Δ*tesA* is unlikely to be sufficient to explain its attenuation.”

Reviewer #3: 1) Fig.4e: The host cell atg1 deletion only partially rescues the ΔtesA mutant! A full rescue means it should grow at the some ate as Mm WT in the Dd Δatg1 cells.

We thank the reviewer for noticing this difference. The difference of Mm Δ*tesA* growth between Dd WT and Dd Δ*atg1* means that Mm WT may still benefit from PDIM action in Dd Δ*atg1*. We agree that a full rescue of Mm Δ*tesA* in Dd Δ*atg1* would have corresponded to a growth similar to Mm WT in Dd Δ*atg1*. We apologise if we were not specific enough in the description of this result. We modified the sentences corresponding to this experiment in the abstract and in the Results section.

Abstract (lines 36-38): “Genetic disruption of host autophagy restored cytosolic access and increased intracellular growth of PDIM-deficient bacteria, indicating that PDIM is specifically required to overcome host repair capacity rather than to initiate damage.”

Results section (lines 378-381): "In contrast, disruption of all autophagy-related pathways, from membrane repair to xenophagy, in Dd Δ*atg1* resulted in a marked increase of Mm Δ*tesA* growth, partially rescuing Mm Δ*tesA* growth compared to Mm WT and in effect restoring Mm Δ*tesA* loads to levels comparable to Mm WT in Dd WT."

2) Fig. 4g/h: The quantification of MmΔtesA on Plin recruitment shows slightsly different results from what was shown in Fig.3c. Why do the authors think this is?

We thank the reviewer for raising this point. We agree that the percentage of Plin-positive bacteria for Mm Δ*tesA* is slightly different between Fig 4h and Fig 3d (previously Fig 3c). First, the two experiments were not performed at exactly the same time points, which could partly explain the difference. Moreover, each experiment was quantified by computer-assisted methods. In Fig 3d (previously Fig 3c), only Dd WT expressing mCherry-Plin were used and the settings were specifically finetuned for this host background. In Fig 4h, both Dd WT and Dd Δ*atg1* were analysed. The settings for the relative measurements were adjusted to quantify the recruitment in both cell lines simultaneously. The settings used for that experiment slightly changed the detection threshold, which slightly alters the absolute levels of Plin-positive Mm detected, but does not influence the relative hierarchy between Mm WT and mutants. In Fig 4h, the higher Plin recruitment was observed not only for Mm Δ*tesA* but also for Mm WT, Mm ΔCE and Mm ΔCEΔ*tesA*, compared with Fig 3d (previously Fig 3c). Although the percentage of Plin recruitment changed, the relative order between the strains was similar between Fig 3d (previously Fig 3c) and Fig 4h and thus, we consider that the results are similar.

1. Guallar-Garrido S, Peret A, Carreras-Sureda A, Michard C, Soldati T. PefA, an ALG-2-like Ca^{2+} sensor, regulates ESCRT and autophagy responses to mycobacterial vacuole damage. bioRxiv. 2026:2026.01.30.702722. doi: 10.64898/2026.01.30.702722.
2. Perret A, Bosmani C, Leuba F, Eblighatian K, Guého A, Raykov L, Hanna N, Soldati T. Membrane microdomains are crucial for Mycobacterium marinum EsxA-dependent membrane damage, escape to the cytosol, and infection. Sci Adv. 2026;12(1):eady0812. Epub 20260101. doi: 10.1126/sciadv.ady0812. PubMed PMID: 41477822; PubMed Central PMCID: PMCPMC12757022.
3. Foulon M, Hüttel SV, Breitsprecher L, Gauda W, Raykov L, Koliwer-Brandl H, Ohlhagen M, Thünauer R, Schwudke D, Hilbi H, Soldati T, Barisch C. Intracellular compartmentalization shapes lipid access and metabolic fitness of mycobacteria. bioRxiv. 2026:2026.02.10.704997. doi: 10.64898/2026.02.10.704997.
4. Raykov L, Mottet M, Nitschke J, Soldati T. A TRAF-like E3 ubiquitin ligase TrafE coordinates ESCRT and autophagy in endolysosomal damage response and cell-autonomous immunity to Mycobacterium marinum. Elife. 2023;12. Epub 20230418. doi: 10.7554/eLife.85727. PubMed PMID: 37070811; PubMed Central PMCID: PMCPMC10181826.
5. Cardenal-Muñoz E, Arafah S, López-Jiménez AT, Kicka S, Falaise A, Bach F, Schaad O, King JS, Hagedorn M, Soldati T. Mycobacterium marinum antagonistically induces an autophagic response while repressing the autophagic flux in a TORC1- and ESX-1-dependent manner. PLoS Pathog. 2017;13(4):e1006344. Epub 20170417. doi: 10.1371/journal.ppat.1006344. PubMed PMID: 28414774; PubMed Central PMCID: PMCPMC5407849.
6. López-Jiménez AT, Cardenal-Muñoz E, Leuba F, Gerstenmaier L, Barisch C, Hagedorn M, King JS, Soldati T. The ESCRT and autophagy machineries cooperate to repair ESX-1-dependent damage at the Mycobacterium-containing vacuole but have opposite impact on containing the infection. PLoS Pathog. 2018;14(12):e1007501. Epub 20181231. doi: 10.1371/journal.ppat.1007501. PubMed PMID: 30596802; PubMed Central PMCID: PMCPMC6329560.
7. Barisch C, Paschke P, Hagedorn M, Maniak M, Soldati T. Lipid droplet dynamics at early stages of Mycobacterium marinum infection in Dictyostelium. Cell Microbiol. 2015;17(9):1332–49. Epub 20150408. doi: 10.1111/cmi.12437. PubMed PMID: 25772333.
8. Koliwer-Brandl H, Knobloch P, Barisch C, Welin A, Hanna N, Soldati T, Hilbi H. Distinct Mycobacterium marinum phosphatases determine pathogen vacuole phosphoinositide pattern, phagosome maturation, and

escape to the cytosol. Cell Microbiol. 2019;21(6):e13008. Epub 20190207. doi: 10.1111/cmi.13008. PubMed PMID: 30656819.
9. Hanson PI, Shim S, Merrill SA. Cell biology of the ESCRT machinery. Curr Opin Cell Biol. 2009;21(4):568–74. Epub 20090626. doi: 10.1016/j.ceb.2009.06.002. PubMed PMID: 19560911; PubMed Central PMCID: PMCPMC6195420.
10. Bernard EM, Fearns A, Bussi C, Santucci P, Peddie CJ, Lai RJ, Collinson LM, Gutierrez MG. infection of human iPSC-derived macrophages reveals complex membrane dynamics during xenophagy evasion. J Cell Sci. 2020;134(5). Epub 20201125. doi: 10.1242/jcs.252973. PubMed PMID: 32938685; PubMed Central PMCID: PMCPMC7710011.
11. Bell SL, Lopez KL, Cox JS, Patrick KL, Watson RO. Galectin-8 Senses Phagosomal Damage and Recruits Selective Autophagy Adapter TAX1BP1 To Control. mBio. 2021;12(4):e0187120. Epub 20210706. doi: 10.1128/mBio.01871-20. PubMed PMID: 34225486; PubMed Central PMCID: PMCPMC8406326.
12. Augenstreich J, Phan AT, Allen CNS, Poddar A, Chen H, Srinivasan L, Briken V. Dynamic interplay of autophagy and membrane repair during Mycobacterium tuberculosis Infection. PLoS Pathog. 2025;21(1):e1012830. Epub 20250102. doi: 10.1371/journal.ppat.1012830. PubMed PMID: 39746091; PubMed Central PMCID: PMCPMC11731705.
13. Chen D, Fearns A, Gutierrez MG. Mycobacterium tuberculosis phagosome Ca^{2+} leakage triggers multimembrane ATG8/LC3 lipidation to restrict damage in human macrophages. Sci Adv. 2025;11(13):eadt3311. Epub 20250326. doi: 10.1126/sciadv.adt3311. PubMed PMID: 40138395; PubMed Central PMCID: PMCPMC11939036.
14. D’Amico D, Goudge G, Foulon M, Macale LS, Prados J, Zheng RB, Franklin A, Lowary TL, Moynihan P, Soldati T. Discoidins are cytosolic lectins that shape intracellular host defence by sensing virulence-associated mycobacterial glycolipids and glycopeptidolipids. bioRxiv. 2026:2026.06.18.733164. doi: 10.64898/2026.06.18.733164.
15. Jia J, Claude-Taupin A, Gu Y, Choi SW, Peters R, Bissa B, Mudd MH, Allers L, Pallikkuth S, Lidke KA, Salemi M, Phinney B, Mari M, Reggiori F, Deretic V. Galectin-3 Coordinates a Cellular System for Lysosomal Repair and Removal. Dev Cell. 2020;52(1):69–87.e8. Epub 20191205. doi: 10.1016/j.devcel.2019.10.025. PubMed PMID: 31813797; PubMed Central PMCID: PMCPMC6997950.
16. Trofimov V, Kicka S, Mucaria S, Hanna N, Ramon-Olayo F, Del Peral LV, Lelièvre J, Ballell L, Scapozza L, Besra GS, Cox JAG, Soldati T. Antimycobacterial drug discovery using Mycobacteria-infected amoebae identifies anti-infectives and new molecular targets. Sci Rep. 2018;8(1):3939. Epub 20180302. doi: 10.1038/s41598-018-22228-6. PubMed PMID: 29500372; PubMed Central PMCID: PMCPMC5834492.
17. Stamm LM, Morisaki JH, Gao LY, Jeng RL, McDonald KL, Roth R, Takeshita S, Heuser J, Welch MD, Brown EJ. Mycobacterium marinum escapes from phagosomes and is propelled by actin-based motility. J Exp Med. 2003;198(9):1361–8. doi: 10.1084/jem.20031072. PubMed PMID: 14597736; PubMed Central PMCID: PMCPMC2194249.
18. Watson RO, Manzanillo PS, Cox JS. Extracellular M. tuberculosis DNA targets bacteria for autophagy by activating the host DNA-sensing pathway. Cell. 2012;150(4):803–15. doi: 10.1016/j.cell.2012.06.040. PubMed PMID: 22901810; PubMed Central PMCID: PMCPMC3708656.
19. Schnettger L, Rodgers A, Repnik U, Lai RP, Pei G, Verdoes M, Wilkinson RJ, Young DB, Gutierrez MG. A Rab20-Dependent Membrane Trafficking Pathway Controls M. tuberculosis Replication by Regulating Phagosome Spaciousness and Integrity. Cell Host Microbe. 2017;21(5):619–28.e5. doi: 10.1016/j.chom.2017.04.004. PubMed PMID: 28494243; PubMed Central PMCID: PMCPMC5432432.
20. Chandra P, Philips JA. USP8 promotes intracellular infection by enhancing ESCRT-mediated membrane repair, limiting xenophagy, and reducing oxidative stress. Autophagy. 2025;21(2):298–314. Epub 20240911. doi: 10.1080/15548627.2024.2395134. PubMed PMID: 39178916; PubMed Central PMCID: PMCPMC11759523.
