# Supplementary material for: PDIM drives the transition from repairable to catastrophic EsxA-mediated vacuole membrane damage during Mycobacterium marinum infection
Source: PLoS Pathog. 2026 Oct 21;22(10):e1014721. doi: 10.1371/journal.ppat.1014721 (PMC13712727; doi:10.1371/journal.ppat.1014721)
Supplement: Supplementary file 3 [file ppat.1014721.s008.docx]

**Table A. Cell lines and bacterial strains used in this study**

| **Strains** | **Relevant characteristics** | **Source / reference** |
| --- | --- | --- |
| *D. discoideum* |  |  |
| Ax2(Ka) | Wild type |  |
| Ax2(Ka) *act5*::GFP-Vps32 |  | [1] |
| Ax2(Ka) *act5*::mCherry-Atg8A |  | [1] |
| Ax2(Ka) *act5*::mCherry-Plin |  | [2] |
| Ax2(Ka) *act6*::GFP-Vps32 *act5*::mCherry-Atg8A |  | This study |
| Ax2(Ka) Δ*vacABC* | Deletion of *vacA*, *vacB*, *vacC* genes | [3] |
| Ax2(Ka) Δ*tsg101* | Deletion of *tsg101* gene | [4] |
| Ax2(Ka) Δ*atg1* | Deletion of *atg1* gene | [5] |
| Ax2(Ka) Δ*atg1* *act5*::mCherry-Plin |  | This study |
| Mammalian cell lines |  |  |
| Murine microglial BV-2 | Wild type |  |
| BV-2 GFP-lysenin |  | This study |
| BV-2 GFP-NDP52 |  | This study |
| BV-2 GFP-LC3b |  | This study |
| HEK 293T | Wild type |  |
| *Mycobacterium* strains |  |  |
| *M. smegmatis mc^2^155* | Phage production |  |
| *M. marinum* M WT | Wild type | L. Ramakrishnan (Washington University) |
| *M. marinum* WT pGFP |  | L. Ramakrishnan (Washington University) |
| *M. marinum* WT pmCherry |  | This study |
| *M. marinum* WT pTec18 |  | This study |
| *M. marinum* ΔRD1 | Deletion of RD1 | L. Ramakrishnan (Washington University) |
| *M. marinum* ΔRD1 pGFP |  | L. Ramakrishnan (Washington University) |
| *M. marinum* ΔCE | Deletion of *esxA*/*esxB*, Kan^R^ | [6] |
| *M. marinum* ΔCE pGFP |  | This study |
| *M. marinum* ΔCE pCherry |  | This study |
| *M. marinum* ΔCE pTec18 |  | This study |
| *M. marinum* ΔCE::CE | Complemented ΔCE mutant, Kan^R^, Apra^R^ | This study |
| *M. marinum* ΔCE::CE pGFP |  | This study |
| *M. marinum* Δ*tesA* | Deletion of *tesA,* Hyg^R^ | This study |
| *M. marinum* Δ*tesA* pGFP |  | This study |
| *M. marinum* Δ*tesA* pCherry |  | This study |
| *M. marinum* Δ*tesA* pEBFP |  | This study |
| *M. marinum* Δ*tesA::tesA* | Complemented Δ*tesA* mutants, Hyg^R^, Apra^R^ | This study |
| *M. marinum* Δ*tesA::tesA* pGFP |  | This study |
| *M. marinum* ΔRD1Δ*tesA* | Deletion of RD1 and *tesA*, Hyg^R^ | This study |
| *M. marinum* ΔRD1Δ*tesA* pGFP |  | This study |
| *M. marinum* ΔCEΔ*tesA* | Deletion of *esxA*/*esxB* and *tesA*, Kan^R^, Hyg^R^ | This study |
| *M. marinum* ΔCEΔ*tesA* | Deletion of *esxA*/*esxB* and *tesA* with unmarked *tesA*, Kan^R^ | This study |
| *M. marinum* ΔCEΔ*tesA* pGFP |  | This study |
| *M. marinum* ΔCEΔ*tesA* pCherry |  | This study |
| *M. marinum* ΔCEΔ*tesA* pTec18 |  | This study |
| *M. marinum* Δ*pks15/1* | *pks15/1* deletion mutant, Hyg^R^ | This study |
| *M. marinum* Δ*pks15/1* pGFP | *pks15/1* mutant expressing GFP, Hyg^R^, Kan^R^ | This study |

**Table B. Primers used in this study**

| Primers | Sequences (5’ …>3’) | Purpose |
| --- | --- | --- |
| oHK47 (tesA-L-Fwd-DraIII) | TTTTTCACAAAGTGCCACATAGACATCGCATTCA | Amplification of upstream AES for *tesA* gene |
| oHK48(tesA-L-Rev- DraIII) | TTTTTCACTTCGTGGTTAGCTTTTCGTCGCTTTT |  |
| oHK49 (tesA-R-Fwd-Van91I) | TTTTTCCATAGATTGGGCTGGTAAGCGACATAGAGA | Amplification of downstream AES for *tesA* gene |
| oHK50 (tesA-R-Rev-Van91I) | TTTTTCCATCTTTTGGCTGCGGTGTGGAAAGTGGGA |  |
| oHK231 (tesA-L-ext) | TTTTTCTACTTACATGTCCCGATTT | Verification of *tesA* gene deletion |
| oHK232 (tesA-R-ext) | TTTTTTGAGCTGGCGTATTTTGTTA |  |
| oHK33 (p0004S-L-fwd) | GGAAGTCAACAAAAAGCAAG | Sequencing primer to verify cloning of correct flanks |
| oHK36 (p0004S-R-rev) | TGGTAGCGGTGGTTTTTTTGT |  |
| 5-PacI-tesA | TGTTAATTAAgTGAACGGGCGCTCAAGC | *tesA* gene to complemente the mutant |
| 3-HindIII-tesA | TTTAAGCTTCTAAATTTGGTCAAACTGCTGC |  |
| 5-HindIII-interG-CE | TTTTTAAGCTTcgtcaacacgaacagacttc | *esxA/B* genes to complemente the mutant |
| 3-ClaI-CE | TTTTATCGATgattaagcaaacatccccgtg |  |
| Pks15/1-up-fd | ATATATGCTCTTCTAGTCGACTGCGGGAATACGAACAA | Amplification of upstream *pks15/1* region |
| Pks15/1-up-rev | TATATATGCTCTTCAAGGTGATCCCGAACGAGGACACAC |  |
| Pks15/1-down-fd | ATATATGCTCTTCTCCTACGGCGAGATGGTTTCGGTA | Amplification of downstream *pks15/1* region |
| Pks15/1-down-rev | TATATAGCTCTTCTTGCCGACTAGCAATCGACTGGGC |  |
| pKO-fd | GGCGATTAAGTTGGGTAACG | To sequence into the pKO vector |
| pKO-rev | GTCATAGCTGTTTCCTTGCG |  |
| pks15/1-up/down-seqfd | GCAACGCAACGGACACAAC | Sequencing primer to verify cloning |
| Pks15/1-up-up-fd | GGCGAATCATGAACAGGAGC | Primers into the genome to verify correct suicide vector insertion |
| Pks15/1-down-down-rev | CATTCCAGCCGCGATACAAG |  |
| pUCoriE-rev | CGATAAGTCGTGTCTTACCG | Primer into OriE region of the suicide vector to verify correct suicide vector insertion |

**Table C. Plasmids used in this study**

| Name (generic name) | Features | Source or Reference |
| --- | --- | --- |
| *D. discoideum* Plasmids |  |  |
| pDM1513-Vps32 | Safe haven knock-in plasmid for the *act5* locus, expression of Vps32 with N-terminal GFP tag, Amp^R^/Hyg^R^ | [1] |
| pDM1515-Atg8A | Safe haven knock-in plasmid for the *act5* locus, expression of Atg8A with N-terminal mCherry tag, Amp^R^/Hyg^R^ | [1] |
| pDM1515-Plin | Safe haven knock-in plasmid for the *act5* locus, expression of Plin with N-terminal mCherry tag, Amp^R^/Hyg^R^ | [2] |
| pDM1515G418-Atg8A | Safe haven knock-in plasmid for the *act5* locus, expression of Atg8A with N-terminal mCherry tag, Amp^R^/Neo^R^ | This study |
| pDMact6-NterGFP-Vps32 | Safe haven knock-in plasmid for the *act6* locus, expression of Vps32 with N-terminal GFP tag, Amp^R^/Hyg^R^ | This study |
| Mammalian plasmids |  |  |
| pMD-VSVG | Packaging vectors to produce retrovirus | gift from F. Randow |
| pMD-OG |  |  |
| pRetro-GFP-lysenin | Amp^R^/Blast^R^ | gift from F. Randow |
| pRetro-GFP-NDP52 | Amp^R^/Blast^R^ | gift from F. Randow |
| pRetro-GFP-LC3b | Amp^R^/Blast^R^ | gift from F. Randow |
| Mycobacterial cloning |  |  |
| p0004S | containing γδres-sacB-hyg-γδres cassette comprising a *sacB* and hygromycin gene flanked by res-sites of γδ-resolvase, Hyg^R^ | [7], W. Jacobs, Einstein College, NY |
| phAE159 | Shuttle phasmid vector for mycobacterial knock-out constructs, derivative of TM4 phage, Amp^R^ | [8],  W. Jacobs, Einstein College, NY |
| pHK12 (p-tesA-S) | *tesA* flanking regions cloned into p0004S vector arms, Hyg^R^ | This study |
| pHK23 (phAE159Δ*tesA*) | Knockout phasmid for *tesA* gene, Hyg^R^ | This study |
| pINIT | Shuttle vector for mycobacterial knock-out constructs, Chl^R^ | [9] |
| pINIT-*pks15/1* | *pks15/1* flanking regions cloned into pINIT vector, Chl^R^ | This study |
| pKOΔ*galK* | Suicide vector for mycobacterial knock-out, Hyg^R^ | Addgene #110090 [9] |
| pKO-*pks15/1* | *pks15/1* flanking regions cloned into pKOΔgalK vector, Hyg^R^ | This study |
| pMV361 | mycobacterial integrative vector for expression under control of the hsp60 promoter, Apr^R^ | [7] |
| pMV361-*tesA* | Complementation plasmid for Δ*tesA* mutant, Apra^R^ | This study |
| pMV361-interG-CE | Complementation plasmid for ΔCE mutant, Apra^R^ | This study |
| pMSP12::GFP | GFP expression under the control of *M. marinum* msp12 promoter, Kan^R^ | gift from L. Ramakrishnan (Addgene #30167) |
| pGFPHYG2 | GFP expression under the control of *M. marinum* msp12 promoter, Hyg^R^ | gift from L. Ramakrishnan (Addgene plasmid # 30173) [10] |
| pCherry10 | mCherry expression under the control of Psmyc promoter, Hyg^R^ | gift from T. Parish (Addgene plasmid # 24664) [11] |
| pMSP12::mCherry | mCherry expression under the control of *M. marinum* msp12 promoter, Kan^R^ | gift from L. Ramakrishnan (Addgene plasmid # 30169) |
| pTec18 | EBFP2 expression under the control of *M. marinum* msp12 promoter, Hyg^R^ | gift from L. Ramakrishnan (Addgene plasmid # 30177) [12] |
| pEBFP | EBFP2 expression under the control of *M. marinum* msp12 promoter, Kan^R^ | This study |

Abbreviations: Amp^R^, ampicillin resistant; Blast^R^, blasticidin resistant ; Chl^R^, chloramphenicol resistant; Hyg^R^, hygromycin resistant; Kan^R^, kanamycin resistant ; Neo^R^, neomycin resistant.

**References**

1.	Raykov L, Mottet M, Nitschke J, Soldati T. A TRAF-like E3 ubiquitin ligase TrafE coordinates ESCRT and autophagy in endolysosomal damage response and cell-autonomous immunity to Mycobacterium marinum. Elife. 2023;12. Epub 20230418. doi: 10.7554/eLife.85727. PubMed PMID: 37070811; PubMed Central PMCID: PMCPMC10181826.

2.	Perret A, Bosmani C, Leuba F, Eblighatian K, Guého A, Raykov L, Hanna N, Soldati T. Membrane microdomains are crucial for Mycobacterium marinum EsxA-dependent membrane damage, escape to the cytosol, and infection. Sci Adv. 2026;12(1):eady0812. Epub 20260101. doi: 10.1126/sciadv.ady0812. PubMed PMID: 41477822; PubMed Central PMCID: PMCPMC12757022.

3.	Bosmani C, Leuba F, Hanna N, Bach F, Burdet F, Pagni M, Hagedorn M, Soldati T. Vacuolins and myosin VII are required for phagocytic uptake and phagosomal membrane recycling in. J Cell Sci. 2020;133(13). Epub 20200706. doi: 10.1242/jcs.242974. PubMed PMID: 32482795.

4.	López-Jiménez AT, Cardenal-Muñoz E, Leuba F, Gerstenmaier L, Barisch C, Hagedorn M, King JS, Soldati T. The ESCRT and autophagy machineries cooperate to repair ESX-1-dependent damage at the Mycobacterium-containing vacuole but have opposite impact on containing the infection. PLoS Pathog. 2018;14(12):e1007501. Epub 20181231. doi: 10.1371/journal.ppat.1007501. PubMed PMID: 30596802; PubMed Central PMCID: PMCPMC6329560.

5.	Cardenal-Muñoz E, Arafah S, López-Jiménez AT, Kicka S, Falaise A, Bach F, Schaad O, King JS, Hagedorn M, Soldati T. Mycobacterium marinum antagonistically induces an autophagic response while repressing the autophagic flux in a TORC1- and ESX-1-dependent manner. PLoS Pathog. 2017;13(4):e1006344. Epub 20170417. doi: 10.1371/journal.ppat.1006344. PubMed PMID: 28414774; PubMed Central PMCID: PMCPMC5407849.

6.	Gao LY, Guo S, McLaughlin B, Morisaki H, Engel JN, Brown EJ. A mycobacterial virulence gene cluster extending RD1 is required for cytolysis, bacterial spreading and ESAT-6 secretion. Mol Microbiol. 2004;53(6):1677–93. doi: 10.1111/j.1365-2958.2004.04261.x. PubMed PMID: 15341647.

7.	Koliwer-Brandl H, Syson K, van de Weerd R, Chandra G, Appelmelk B, Alber M, Ioerger TR, Jacobs WR, Geurtsen J, Bornemann S, Kalscheuer R. Metabolic Network for the Biosynthesis of Intra- and Extracellular α-Glucans Required for Virulence of Mycobacterium tuberculosis. PLoS Pathog. 2016;12(8):e1005768. Epub 20160811. doi: 10.1371/journal.ppat.1005768. PubMed PMID: 27513637; PubMed Central PMCID: PMCPMC4981310.

8.	Bardarov S, Kriakov J, Carriere C, Yu S, Vaamonde C, McAdam RA, Bloom BR, Hatfull GF, Jacobs WR. Conditionally replicating mycobacteriophages: a system for transposon delivery to Mycobacterium tuberculosis. Proc Natl Acad Sci U S A. 1997;94(20):10961–6. doi: 10.1073/pnas.94.20.10961. PubMed PMID: 9380742; PubMed Central PMCID: PMCPMC23545.

9.	Arnold FM, Hohl M, Remm S, Koliwer-Brandl H, Adenau S, Chusri S, Sander P, Hilbi H, Seeger MA. A uniform cloning platform for mycobacterial genetics and protein production. Sci Rep. 2018;8(1):9539. Epub 20180622. doi: 10.1038/s41598-018-27687-5. PubMed PMID: 29934571; PubMed Central PMCID: PMCPMC6015033.

10.	Cosma CL, Klein K, Kim R, Beery D, Ramakrishnan L. Mycobacterium marinum Erp is a virulence determinant required for cell wall integrity and intracellular survival. Infect Immun. 2006;74(6):3125–33. doi: 10.1128/IAI.02061-05. PubMed PMID: 16714540; PubMed Central PMCID: PMCPMC1479242.

11.	Carroll P, Schreuder LJ, Muwanguzi-Karugaba J, Wiles S, Robertson BD, Ripoll J, Ward TH, Bancroft GJ, Schaible UE, Parish T. Sensitive detection of gene expression in mycobacteria under replicating and non-replicating conditions using optimized far-red reporters. PLoS One. 2010;5(3):e9823. Epub 20100323. doi: 10.1371/journal.pone.0009823. PubMed PMID: 20352111; PubMed Central PMCID: PMCPMC2843721.

12.	Takaki K, Davis JM, Winglee K, Ramakrishnan L. Evaluation of the pathogenesis and treatment of Mycobacterium marinum infection in zebrafish. Nat Protoc. 2013;8(6):1114–24. Epub 20130516. doi: 10.1038/nprot.2013.068. PubMed PMID: 23680983; PubMed Central PMCID: PMCPMC3919459.
