# Supplementary material for: PDIM drives the transition from repairable to catastrophic EsxA-mediated vacuole membrane damage during Mycobacterium marinum infection
Source: PLoS Pathog. 2026 Oct 21;22(10):e1014721. doi: 10.1371/journal.ppat.1014721 (PMC13712727; doi:10.1371/journal.ppat.1014721)
Supplement: Peer review history

# Peer review: Decision Letter 0

**DOI:** 10.1371/journal.ppat.1014721.r001

**Authors:** Michael Otto, Pierre Santucci

17 Aug 2026

PPATHOGENS-D-26-01539

PDIM drives the transition from repairable to catastrophic EsxA-mediated vacuole membrane damage during *Mycobacterium marinum*  infection

PLOS Pathogens

Dear Dr. Soldati,

Thank you for submitting your manuscript to PLOS Pathogens. After careful consideration, we feel that it has merit but does not fully meet PLOS Pathogens's publication criteria as it currently stands. Therefore, we invite you to submit a revised version of the manuscript that addresses the points raised during the review process.

Please submit your revised manuscript by Sep 16 2026 11:59PM. If you will need more time than this to complete your revisions, please reply to this message or contact the journal office at plospathogens@plos.org. When you're ready to submit your revision, log on to https://www.editorialmanager.com/ppathogens/ and select the 'Submissions Needing Revision' folder to locate your manuscript file.

Please include the following items when submitting your revised manuscript:

* A letter that responds to each point raised by the editor and reviewer(s). You should upload this letter as a separate file labeled 'Response to Reviewers'. This file does not need to include responses to any formatting updates and technical items listed in the 'Journal Requirements' section below.

* A marked-up copy of your manuscript that highlights changes made to the original version. You should upload this as a separate file labeled 'Revised Manuscript with Track Changes'.

* An unmarked version of your revised paper without tracked changes. You should upload this as a separate file labeled 'Manuscript'.

If you would like to make changes to your financial disclosure, competing interests statement, or data availability statement, please make these updates within the submission form at the time of resubmission. Guidelines for resubmitting your figure files are available below the reviewer comments at the end of this letter.

As the corresponding author, your ORCID iD is verified in the submission system and will appear in the published article. PLOS supports the use of ORCID, and we encourage all coauthors to register for an ORCID iD and use it as well. Please encourage your coauthors to verify their ORCID iD within the submission system before final acceptance, as unverified ORCID iDs will not appear in the published article. Only the individual author can complete the verification step; PLOS staff cannot verify ORCID iDs on behalf of authors.

We look forward to receiving your revised manuscript.

Kind regards,

Pierre Santucci

Academic Editor

PLOS Pathogens

Michael Otto

Section Editor

PLOS Pathogens

Sumita Bhaduri-McIntosh

Editor-in-Chief

PLOS Pathogens

orcid.org/0000-0003-2946-9497

Dominique Soldati-Favre

Editor-in-Chief

PLOS Pathogens

orcid.org/0000-0003-4156-2109

**Additional Editor Comments (if provided):**

Dear Thierry,

Thanks for submitting your work to PLOS Pathogens. As you will see, the three independent experts have now carefully inspected your work and found it of great quality.

Nevertheless, they raised some concerns that might need some changes, including text modification and re-editing of the manuscript. Their comments are available attached to this decision letter. Some concerns raised by Reviewer 1 and 3 might require additional experimental work, however should you feel that this is not justified to support your claims, please carefully address this in your point by point response.

I look forward to receiving your revised manuscript.

Thanks again for choosing PLOS Pathogens for your work.

Sincerely,

Pierre

**Journal Requirements:**

1. Please ensure that the CRediT author contributions listed for every co-author are completed accurately and in full.

At this stage, the following Authors require contributions: Thierry Soldati. Please ensure that the full contributions of each author are acknowledged in the "Add/Edit/Remove Authors" section of our submission form.

The list of CRediT author contributions may be found here: https://journals.plos.org/plospathogens/s/authorship#loc-author-contributions

2. Please amend your detailed Financial Disclosure statement. This is published with the article. It must therefore be completed in full sentences and contain the exact wording you wish to be published.

1. State what role the funders took in the study. If the funders had no role in your study, please state: “The funders had no role in study design, data collection and analysis, decision to publish, or preparation of the manuscript.”

2. If any authors received a salary from any of your funders, please state which authors and which funders.

If you did not receive any funding for this study, please simply state: “The authors received no specific funding for this work.”

3. We ask that a manuscript source file is provided at Revision. Please upload your manuscript file as a .doc, .docx, .rtf or .tex.

4. Please provide separate figure files in .tif or .eps format.

For more information about figure files please see our guidelines: https://journals.plos.org/plospathogens/s/figures

https://journals.plos.org/plospathogens/s/figures#loc-file-requirements

5. We do not publish any copyright or trademark symbols that usually accompany proprietary names, eg (R), (C), or TM  (e.g. next to drug or reagent names). Please remove all instances of trademark/copyright symbols throughout the text, including "CF®405M" on page 32.

6. PLOS now requires that authors provide the original uncropped and unadjusted images underlying all blot or gel results reported in a submission’s figures or Supporting Information files. This policy and the journal’s other requirements for blot/gel reporting and figure preparation are described in detail at https://journals.plos.org/plospathogens/s/figures#loc-blot-and-gel-reporting-requirements and https://journals.plos.org/plospathogens/s/figures#loc-preparing-figures-from-image-files.

When you submit your revised manuscript, please ensure that your figures adhere fully to these guidelines and provide the original underlying images for all blot or gel data reported in your submission. See the following link for instructions on providing the original image data: https://journals.plos.org/plospathogens/s/figures#loc-original-images-for-blots-and-gels.

In your cover letter, please note whether your blot/gel image data are in Supporting Information or posted at a public data repository, provide the repository URL if relevant, and provide specific details as to which raw blot/gel images, if any, are not available. Email us at customercare@plos.org if you have any questions.

7. We have noticed that you have uploaded Supporting Information Tables, but you have not included a list of legends. Please add a full list of legends for your Supporting Information files after the references list.

8. We note that Figure 6 is created through BioRender. Please confirm that you hold a Premium account and provide a pdf copy of the CC BY 4.0 Licence as provided by BioRender. For instructions on how to generate a CC BY 4.0 license for your figure, please see the guidelines here: https://help.biorender.com/hc/en-gb/articles/21282341238045-How-to-download-a-publication-license-for-open-access-resources.

Please ensure the figure legend contains appropriate attribution text & a link to biorender.com

If you are using the free assets from BioRender, we are unable to publish these images as they are licenced under a stricter licence than CC BY 4.0. In this case we ask you to remove the BioRender images and replace them with open source alternatives.

See these open source resources you may use to replace images / clip-art:

- https://bioart.niaid.nih.gov/

- https://bioicons.com/

- https://healthicons.org/

- https://scidraw.io/

- https://reactome.org/icon-lib

- https://www.phylopic.org/images

- https://journals.plos.org/plosbiology/article?id=10.1371/journal.pbio.3002395

If the reviewer comments include a recommendation to cite specific previously published works, please review and evaluate these publications to determine whether they are relevant and should be cited. There is no requirement to cite these works unless the editor has indicated otherwise.

[Note: HTML markup is below. Please do not edit.]

**Reviewers' Comments:**

Reviewer's Responses to Questions

**Part I - Summary**

Please use this section to discuss strengths/weaknesses of study, novelty/significance, general execution and scholarship.

Reviewer #1: The manuscript by Michard et al is a great piece of work that attempts to decipher the respective contributions of ESX-1 effectors and the PDIM lipids during infection by Mycobacterium marinum. The authors succesfully use host reporters in two infection models to show how these factors act at different stages of the dynamic process of damage and rupture of the MCV. Overall the work is solid, although I have a few major points that could be improved to ensure the strength of the work. Importantly, from a technical point of view, given that they are trying to show co-localization with different cell markers, it becomes indispensable that the authors change their images to colour-blind safe combinations, I have also stated this under Minor issues.

I think for the field, this manuscript clearly shows how there is a difference between EsxA-induced damage that is amplified by PDIM, which is a key knowledge gap. I think further dissection of how it works mechanistically is something that can be tackled in the future.

Reviewer #2: This manuscript thoroughly addresses an important open question regarding the contribution of PDIM to membrane damage during Mm infection. The authors use genetic perturbations in both the bacteria and the host to investigate the sequential contributions of EsxA and PDIM to cytosolic access by Mm. The manuscript is clearly written and appropriately references recent literature, providing a detailed overview of the current understanding in the field and clearly explaining how this study contributes to resolving several outstanding questions. A few minor improvements could further enhance clarity for readers in certain sections, as outlined in the minor comments below.

Reviewer #3: The study by Michard et al demonstrates the synergy between EsxA/B and PDIM-driven membrane damage. The authors use state-of-the-art live cell imaging approaches in combination with an extensive series of Mm deletion mutants and/or host cell mutants to show the differential impact of the lack of EsxA/B, PDIM/PGL or both on host cell ESCRT and autophagy responses. Overall the work significantly advances the knowledge in the field generated by the Soldati lab in the Dictyo/Mm model and other labs in the Mtb/macrophage model.

Major concerns/critiques:

1) There seems to be a discrepancy between the growth of the Mm ∆tesA mutant in Dd (Fig. 1d) and BV2 cells (Fig. 5b) which needs to be addressed

2) There is strong evidence that ER-mediated membrane repair is of particular importance for membrane repair after infection with either Mtb or Mm. How the mutants affect recruitment of ER-membrane repair proteins to the MCV should be included to complete the picture

Minor:

1) Fig.4e: The host cell atg1 deletion only partially rescues the ∆tesA mutant! A full rescue means it should grow at the some ate as Mm WT in the Dd ∆atg1 cells.

2) Fig. 4g/h: The quantification of Mm∆tesA on Plin recruitment shows slightsly different results from what was shown in Fig.3c. Why do the authors think this is?

**********

**Part II – Major Issues: Key Experiments Required for Acceptance**

Please use this section to detail the key new experiments or modifications of existing experiments that should be absolutely required to validate study conclusions.

Generally, there should be no more than 3 such required experiments or major modifications for a "Major Revision" recommendation. If more than 3 experiments are necessary to validate the study conclusions, then you are encouraged to recommend "Reject".

Reviewer #1: The first main issue I see with the manuscript in its current version is the use of diverse host markers, especially for damage and rupture and later on, autophagy, but they never show co-localization with a MCV marker, making me question how can the authors truly differentiate between cytosolic and vacuolar Mm.

Second, it is well established in the field of membrane damage and rupture during bacterial pathogenesis, that a good marker for rupture is lysenin, which the authors use; but for rupture they did not use any of the established rupture markers, such as galectin (3 or 8) which are crucial to differentiate damaged vacuoles (lysenin positive, galectin negative) from ruptured vacuoles (galectin positive). Such experiments I would consider crucial to differentiate between the two stages and would validate their Plin experiments, as this only binds the myco cell wall but does not report for the MCV membrane itself.

In addition, the authors could complement their plate reader experiments with CFU counting, especially considering that they are not tracking individual bacteria overtime by microscopy.

Reviewer #2: (No Response)

Reviewer #3: 1) There seems to be a discrepancy between the growth of the Mm ∆tesA mutant in Dd (Fig. 1d) and BV2 cells (Fig. 5b) which needs to be addressed

2)There is strong evidence that ER-mediated membrane repair is of particular importance for membrane repair after infection with either Mtb or Mm. How the mutants affect recruitment of ER-membrane repair proteins to the MCV should be included to complete the picture

**********

**Part III – Minor Issues: Editorial and Data Presentation Modifications**

Please use this section for editorial suggestions as well as relatively minor modifications of existing data that would enhance clarity.

Reviewer #1: 1) I think it is fundamental for the authors to change their figures to a colour blind-safe combination, and avoid all green-red combinations to show co-localizations. This is especially critical for figures with low magnification like 1c, 2g, etc, but it should be implemented in all of them.

2) It is interesting to this reviewer that in Figure 3c there seems to be a fixed percentage of bacteria accessing the cytosol up to 36 hpi. Can the authors check if there is replication in the cytosol? Is this the total subpopulation that escapes to the cytosol?

3) Regarding data presentation, it would be ideal to include the time post infection in all figure panels in which representative microscopy images are presented. I detected that in some figure legends this is not stated, such as 2a-b-c-d-e-f, 3a-b-c, 4g, 5c-d-e-f (here the authors state that cells were fixed at 5 and 24 hpi, but it is not stated which time point is shown in the selected images).

4) In line 432, the authors state “Ubiquitin accumulation is an early sign of membrane damage and persist until the compartment is repaired or cleared”, please provide the corresponding citation.

5) I found Figure S3 quite confusing, although is supposed to show individual tracks of MCVs overtime, some of them are merged. It would make much more sense to keep them individual. Moreover, for MCVs tracked that are appearing and disappearing from the field of view (from what I can interpret), how did the authors ensured that they are from the same cells?

Reviewer #2: For the experiments shown in Figures 1d and 5b, why did the measurements end at 48h rather than 72h? Was this due to a technical limitation of the imaging approach, or was the 48h endpoint selected intentionally? If so, the rationale for changing the endpoint should be clarified. Although extending the experiment may not alter the overall conclusion, it could further emphasize the observed differences and allow a more direct comparison with Figure 1b.

In Figure 1c, the micrographs are difficult to interpret/see, particularly the smaller grayscale images. Including representative zoom ins would help readers to better understand the differences between conditions. More generally, for all colocalization images, it would be helpful to display the individual channels separately, as shown in Figures 2a–c, and to include zoom ins centered on the bacteria. Using a color-blind-friendly colors and avoiding gray where possible would further improve.

In Figure 2, the authors show representative examples of the dynamic recruitment patterns of Vps32 and Atg8 to the WT and mutant Mm strains. It would be helpful to include a schematic summarizing these distinct recruitment events and grouping them according to their temporal patterns, either alongside the corresponding images or as an additional panel within the figure. The figure would also benefit from indicating the frequency of each event and clearly linking these categories to the quantification shown in Supplementary Figure 3. Although Supplementary Figure 3 contains multiple examples, it is currently difficult to relate them directly to the representative events shown in Figure 2. One possible solution would be to include the corresponding timeline bars from Supplementary Figure 3 beneath each representative example.

For Figures 3 and 4, again a graphical summary linking the different damage and repair markers to the authors’ conclusions would help readers follow the proposed sequence of events. The authors could adapt the model presented in Figure 6 to illustrate Vps32, Atg8 and Plin recruitment as an indicator the damage stage. Including a schematic alongside these figures would make the interpretation of the different markers and their relationship to the reader more intuitive.

The authors state in line 348: “Importantly, the reduction in MCV damage resulted in confinement of Mm WT and thus phenocopied infection with the Mm ΔRD1 mutant.” It would be helpful to specify which data support this comparison and to include the corresponding figure reference at the end of the text.

One last concern is the level of EsxA secretion in the tesA mutant. Although the authors acknowledge this issue in the limitations section, it would be helpful to discuss it earlier. In particular, the authors could compare the tesA mutant with the complemented CE strain, which also appears to exhibit reduced EsxA secretion despite phenotypic complementation. Discussing the distinct damage/repair profiles observed between the wild-type, tesA and CE strain would strengthen the interpretation of the results.

Reviewer #3: 1) Fig.4e: The host cell atg1 deletion only partially rescues the ∆tesA mutant! A full rescue means it should grow at the some ate as Mm WT in the Dd ∆atg1 cells.

2) Fig. 4g/h: The quantification of Mm∆tesA on Plin recruitment shows slightsly different results from what was shown in Fig.3c. Why do the authors think this is?

**********

PLOS authors have the option to publish the peer review history of their article (what does this mean?). If published, this will include your full peer review and any attached files.

If you choose “no”, your identity will remain anonymous but your review may still be made public.

**Do you want your identity to be public for this peer review?**  For information about this choice, including consent withdrawal, please see our Privacy Policy.

Reviewer #1: No

Reviewer #2: No

Reviewer #3: No

[NOTE: If reviewer comments were submitted as an attachment file, they will be attached to this email and accessible via the submission site. Please log into your account, locate the manuscript record, and check for the action link "View Attachments". If this link does not appear, there are no attachment files.]

**Figure resubmission:**

**Reproducibility:**

To enhance the reproducibility of your results, we recommend that authors of applicable studies deposit laboratory protocols in protocols.io, where a protocol can be assigned its own identifier (DOI) such that it can be cited independently in the future. Additionally, PLOS ONE offers an option to publish peer-reviewed clinical study protocols. Read more information on sharing protocols at https://plos.org/protocols?utm_medium=editorial-email&utm_source=authorletters&utm_campaign=protocols

# Author comment: Author response to Decision Letter 1

**DOI:** 10.1371/journal.ppat.1014721.r002

28 Sep 2026

Attachment Submitted filename: Response to Reviewers.pdf

# Editor report: Decision Letter 1

**DOI:** 10.1371/journal.ppat.1014721.r003

**Authors:** Michael Otto, Pierre Santucci

6 Oct 2026

Dear Dr. Soldati,

We are pleased to inform that your manuscript, "PDIM drives the transition from repairable to catastrophic EsxA-mediated vacuole membrane damage during *Mycobacterium marinum*  infection", has been editorially accepted for publication at PLOS Pathogens.

Before your manuscript can be formally accepted and sent to production, you will need to complete our formatting changes, which you will receive by email within a week. Please note that your manuscript will not be scheduled for publication until you have made the required changes.

IMPORTANT NOTES

(1) Copyediting and Proofreading: The corresponding author will receive a typeset proof for review, to ensure errors have not been introduced during production. Please review the PDF proof of your manuscript carefully, as this is the last chance to correct any errors. Please note that major changes, or those which affect the scientific understanding of the work, will likely cause delays to the publication date of your manuscript.

(2) Appropriate Figure Files: Please remove all name and figure # text from your figure files. Please also take this time to check that your figures are of high resolution, which will improve the readbility of your figures and help expedite your manuscript's publication. Please note that figures must have been originally created at 300dpi or higher. Do not manually increase the resolution of your files. For instructions on how to properly obtain high quality images, please review our Figure Guidelines, with examples at: http://journals.plos.org/plospathogens/s/figures.

We encourage you to use PACE (http://pace.apexcovantage.com/), a figure-checking tool which helps authors ensure all figures meet PLOS requirements so that figures can publish in the highest quality. PACE is a digital diagnostic and conversion tool for figure files. It will provide information about any failed check(s) and, if able, will automatically convert the figure into an acceptable file that passes quality checks.

To use PACE, you must first register as a user. Then, login and navigate to the UPLOAD tab, where you will find detailed instructions on how to use the tool.

(3) Striking Image: Please upload a striking still image to accompany your article if one is available (you can include a new image or an existing one from within your manuscript). Should your paper be accepted, this image will be considered for our monthly issue image and may also appear on our website to feature your article. Please upload this as a separate file, selecting "striking image" as the file type upon upload. Please also include a separate "Other" file with a caption, including credits and any potential copyright information. Please do not include the caption in the main article file. If your image is from someone other than yourself, please ensure that the artist has read and agreed to the terms and conditions of the Creative Commons Attribution License at http://journals.plos.org/plospathogens/s/content-license. Please note that PLOS cannot publish copyrighted images.

(4) Press Release or Related Media: If you or your institution will be preparing press materials for this manuscript, please inform our press team in advance at plospathogens@plos.org as soon as possible. We ask that you contact us within one week to plan ahead of our fast Production schedule. If you need to know your paper's publication date for related media purposes, you must coordinate with our press team, and your manuscript will remain under a strict press embargo until the publication date and time.

(5) Update your Profile Information: Now that your manuscript has been provisionally accepted, please log into Editorial Manager and update your profile, if needed. Go to http://www.editorialmanager.com/ppathogens, log in, and click on the "Update My Information" link at the top of the page. Please update your user information to ensure an efficient production and billing process.

(6) LaTeX users only: Our staff will ask you to upload a TEX file in addition to the PDF before the paper can be sent to typesetting, so please carefully review our Latex Guidelines http://journals.plos.org/plospathogens/s/latex in the meantime.

Thank you again for supporting Open Access publishing; we are looking forward to publishing your work in PLOS Pathogens.

Best regards,

Pierre Santucci

Academic Editor

PLOS Pathogens

Michael Otto

Section Editor

PLOS Pathogens

Dominique Soldati-Favre

Editor-in-Chief

PLOS Pathogens

***********************************************************

Dear Dr. Soldati,

After evaluation of your responses to the experts and careful analysis of the revised version of the manuscript, the editorial board is pleased to accept you manuscript for publication in PLOS Pathogens.

Thank you again for submitting your work and contributing to PLOS Pathogens.

Sincerely,

Pierre

# Editor report: Acceptance letter

**DOI:** 10.1371/journal.ppat.1014721.r004

**Authors:** Michael Otto, Pierre Santucci

Dear Dr. Soldati,

We are delighted to inform you that your manuscript, "PDIM drives the transition from repairable to catastrophic EsxA-mediated vacuole membrane damage during *Mycobacterium marinum*  infection," has been formally accepted for publication in PLOS Pathogens.

We have now passed your article onto the PLOS Production Department who will complete the rest of the pre-publication process. All authors will receive a confirmation email upon publication.

Thank you again for supporting open-access publishing; we are looking forward to publishing your work in PLOS Pathogens.

Best regards,

Dominique Soldati-Favre

Editor-in-Chief

PLOS Pathogens
